# Regulation of VEGFR2 trafficking and signaling by Rab GTPase-activating proteins

**DOI:** 10.1038/s41598-019-49646-4

**Published:** 2019-09-16

**Authors:** Ye Xie, Maysam Mansouri, Aurélien Rizk, Philipp Berger

**Affiliations:** 1 Paul Scherrer Institute, Laboratory of Nanoscale Biology, CH-5232 Villigen, PSI Switzerland; 2 Present Address: ETH Zurich, Department of Biosystems Science and Engineering, CH-4058 Basel, Switzerland; 3 Present Address: InterAx Biotech AG, CH-5232 Villigen, PSI Switzerland

**Keywords:** Cell signalling, Cancer

## Abstract

Vascular endothelial growth factor receptor-2 (VEGFR2) and its ligands (VEGFs) are crucial players in vasculogenesis and angiogenesis. General blocking of this signaling system with antibodies or small molecule inhibitors is an established strategy to treat cancer and age-related macular degeneration. Nevertheless, the activated receptor can signal to discrete downstream signaling pathways and the equilibrium between these pathways is modulated by coreceptors and distinct isoforms of VEGF. Here we investigated the influence of Rab GTPase activating proteins (RabGAPs) on VEGFR2 signaling, tube formation, and migration of endothelial cells. We demonstrate that members of the TBC1D10 subfamily of RabGAPs have opposite effects. Whereas TBC1D10A leads to increased Erk1/2 signaling, TBC1D10B lowered Erk1/2 and p38 signaling and reduced tube formation *in vitro*. TBC1D10A is a RabGAP acting on RAB13 that was shown before to play a role in angiogenesis and we could indeed show colocalization of these two proteins with VEGFR2 in activated cells. In addition, we observed that cells expressing TBC1D10B show lower expression of VEGFR2 and NRP1 on filopodia of activated cells. Taken together, our systematic analysis of influence of RabGAPs on VEGFR2 signaling identifies the TBC1D10 subfamily members as modulators of angiogenesis.

## Introduction

Vasculogenesis, angiogenesis, and lymphangiogenesis are essential processes during development, regeneration, and certain pathological conditions in multicellular organisms. Vascular endothelial growth factor receptors (VEGFRs) are key players in these processes. VEGFR-1/-2/-3 form a subfamily in the large family of receptor tyrosine kinases (RTKs). They consist of seven extracellular Ig domains, a single transmembrane spanning region, and an intracellular kinase domain. Whereas VEGFR2 and VEGFR3 belong to the main drivers of angiogenesis and lymphangiogenesis, VEGFR1 has only a weak signaling potential. Its extracellular part also exists in a soluble form indicating that this receptor plays an important role as a decoy receptor for VEGF signaling. The importance of VEGFRs is underscored by the fact that deletion of all three VEGFR genes in mice lead to embryonic lethality^1–3^. VEGFRs are activated by vascular endothelial growth factors (VEGFs). There are five VEGF genes in the human genome (VEGF-A/-B/-C/-D and placental growth factor (PlGF)) all coding for proteins with approximately 100 to 200 amino acids that exist as dimers *in vivo*. Alternative splicing of mRNAs or proteolytic cleavage of all five VEGFs leads to a wide range of isoforms with distinct functions that bind in an overlapping manner to the three VEGFRs. Binding of VEGF to VEGFRs leads to homo- or heterodimerization of the receptors, followed by autophosphorylation of the cytoplasmic domains of VEGFRs^4^.

Several tyrosine and serine phosphorylation sites have been identified in VEGFR2 that link to downstream signaling pathways or degradation. These phosphorylated tyrosine residues recruit specific adaptor proteins. For example, phosphorylated Y949 binds to TSAd that then activates c-Src at adherens junctions, leading to a transient weakening of these junctions^5^. Phosphorylated Y1175 binds to phospholipase Cγ (PLCγ), leading to activation of Erk1/2 pathways, and is important for proliferation and differentiation of endothelial cells during development^6^. Phosphorylation of Y1212 recruits a Nck/ fyn complex that is involved in the activation of cdc42 and p38. Mutation of this site to a phenylalanine in transgenic mice is in contrast to other tyrosine phosphorylation sites not lethal^7^. Serine phosphorylation at a PEST motif of VEGFR2 leads to the recruitment of the E3 ubiquitin ligase β-Trcp1 followed by polyubiquitination and degradation of VEGFR2^8^.

Originally it was thought that signaling of RTKs occurs exclusively at the plasma membrane and that endocytosis of the activated receptor is only needed for degradation of the receptor. There are now several lines of evidence showing that the intracellular trafficking and localization of VEGFR2 is important for signaling. Trafficking and localization of VEGFRs is regulated by available coreceptors in a cell type-specific manner and by the presence of different isoforms of VEGF in the extracellular environment^9^. In tip cells of developing vessel sprouts, VEGFR2 is rapidly activated and internalized, whereas it is co-localized with vascular endothelial-phosphotyrosine phosphatase (VE-PTP) in neighboring stalk cells leading to reduced signaling^10^. Neuropilin-1 (NRP1), initially identified as receptor for class-3 semaphorins, is another coreceptor of VEGFR2. NRP1 is linked to VEGFR2 by VEGF-A. Interestingly, not all isoforms of VEGF-A are able to recruit NRP1. Binding depends on a C-terminal motif of VEGF-A that only exists in a-type isoforms, e.g. VEGF-A165a^11^. Recruitment of NRP1 then leads to recycling through Rab11 recycling vesicles and increased p38 signaling^12^. Nevertheless, the situation is more complex, since VEGF-A165a can also interact with NRP1 on neighboring cells^13^. Ephrin-B2 is another membrane protein that controls angiogenesis together with VEGFRs. They are linked together by the cytoplasmic proteins Disabled 2 (Dab2), clathrin-associated sorting protein (CLASP)17, and the cell polarity protein PAR-3. Endocytosis and signaling of this complex is controlled by the activity of aPKC, which is high in established vessels but low in endothelial sprouts^14^. Contact with cytoplasmic phosphatases also has an influence on signaling. Protein tyrosine phosphatase-1B (PTP1B) is a cytoplasmic phosphatase that negatively regulates arteriogenesis^15^. Mutations or deletions of myosin-VI, GIPC, or NRP1 lead to a delayed trafficking of VEGFR2 through early endosomes, giving PTP1B more time to dephosphorylate VEGFR2 thus leading to reduced Erk1/2 signaling^16^. It is therefore relevant to know the localization of VEGFR2 within a cell.

Rab GTPases are small GTPase that localize to specific intracellular compartments where they regulate cargo trafficking. They are therefore excellent markers to monitor trafficking of RTKs. It was shown before that VEGFR2 colocalizes with several Rab GTPases and that these colocalizations are linked to signaling events.

Endocytosed VEGFR2 rapidly goes into early endosomes (EE) colocalizing with Rab5^17^. The recycling of VEGFR2 to the plasma membrane proceeds via Rab4 to Rab11-associated vesicles, a so-called long-loop recycling, which has a half-time of approximately 15 to 30 minutes^18^. VEGFR2 can also move to vesicles colocalizing with Rab7, a GTPase governing early-to-late endosomal maturation, then subsequently be degraded in lysosomes or proteasomes^19^. In the absence of coreceptor NRP1, most of the VEGFR2 population is diverted to Rab7-associated late endosomes and undergoes degradation. However when co-trafficked with NRP1, the recycled VEGFR2 fraction is increased and the pro-angiogenic phosphorylation of p38 increases as well^12^. Rab13 is another Rab GTPases involved in VEGFR2 trafficking. VEGF-A stimulation recruits a protein complex of RhoA with its GEF Syx. This complex translocates to Rab13-positive vesicles and moves to the leading edge of migrating cells^20^.

Rab GTPases can be converted to their inactive state by RabGAPs. They can therefore be used to inactivate Rab GTPases, thereby interfering with the transport processes these Rabs are involved in. The human genome contains 39 RabGAPs, and overexpression of these RabGAPs was used before to analyze the trafficking of Shiga toxin, the uptake of EGF, and the processing of amyloid precursor protein (APP)^21,22^. We used this library to get a better insight into the influence of vesicular trafficking on VEGFR2 signaling. VEGFR2 is an important target in the pharmaceutical industry for treatment of cancer and macular degeneration, and is currently only addressed by general inhibitors, e.g. with antibodies (e.g. bevacizumab, ranibizumab) or RTK inhibitors (e.g. pazopanib, regorafenib). Nevertheless, long term treatment of patients has adverse effect such as hypertension or delayed wound healing^23^. Increased insights into localization of VEGFR2 signaling can permit the development of drugs that specifically suppress certain pathways. For example, it could be interesting to specifically inhibit signal transduction pathways responsible for deterioration of vessel function in pathologies while preserving vessel survival.

We used a previously developed library of RabGAPs to address their influence on intracellular signaling transmission and the capacity for angiogenesis. We identified TBC1D10A-C as regulators of VEGFR2 and tube formation of human umbilical vein endothelial cells (HUVEC). Interestingly, these three TBC1D10 proteins have opposite effects in signaling and tube formation.

## Results

### Lentivirus-based expression of RabGAPs in endothelial cells

**Figure 1:**
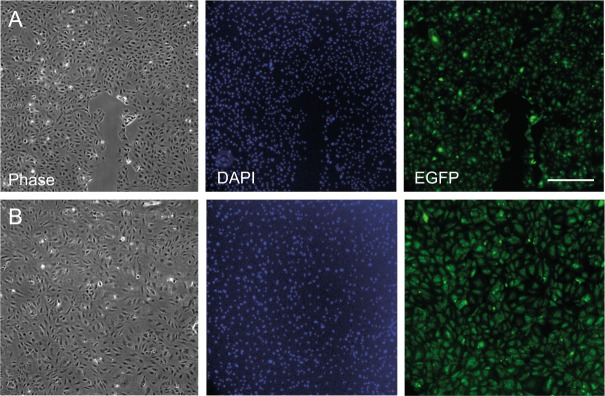
Efficient expression of RabGAPs in primary cells. HUVEC were transduced with purified and concentrated lentivirus encoding (**A**) EGFP only as a control and (**B**) EGFP-TBC1D22B. Images show that HUVEC are efficiently transduced by lentivirus and that a homogenous cell population can be obtained. Scale bar: 500 μm.

The RabGAP library was developed by Fuchs *et al*. to investigate Shiga toxin and EGF uptake^21^. In this library 39 RabGAPs were cloned with an N-terminal EGFP tag in the pEGFP-C2 vector (CLONTECH Laboratories, Inc.). Clones were transiently transfected into HeLa cells, a cell line that is easy to transfect^21^. Primary endothelial cells are difficult to transfect, and we needed high transduction efficiency for our experiments. We therefore transferred the coding regions of all EGFP-RabGAPs in a lentivirus backbone vector^24^. The sizes of the EGFP-RabGAPs range between 3 to 6 kbp. Lentivirus has a limited DNA capacity for foreign genes and some RabGAPs with relatively large coding regions (around 5 kbp) were packaged with less efficiency. Concentrated and purified virus was used to increase infection efficiency and to decrease toxicity. We were able to express 33 of 39 RabGAPs with this strategy in HUVEC and PAE cells with high efficiency (>90%). The transduction efficiency could be measured by fluorescence microscopy since the transduced cells express EGFP-RabGAP fusion protein (Fig. 1). We failed to express e.g. TRE17/Ubiquitin-specific Protease 6 (USP6) probably due to its long cDNA (6 kbp). The lentivirus-based version of the RabGAP library should be a useful tool for other experiments that depend on primary cells or cells that are difficult to transfect.

### Modulation of VEGFR2 signaling by RabGAPs

**Figure 2:**
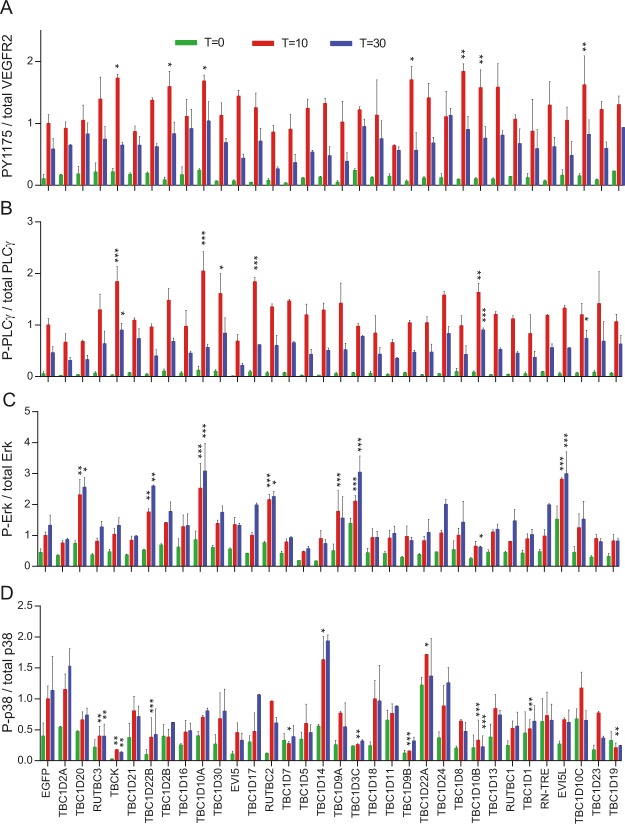
Influence of RabGAP overexpression on VEGFR2 signaling. PAE cells stably expressing VEGFR2 and NRP1 were transduced with lentivirus expressing RabGAPs and then stimulated with VEGF-A165a for 0, 10 and 30 minutes. Stimulation after 10 minutes of EGFP transduced cells was set to 1. *Denotes p < 0.01, **p < 0.005, ***p < 0.001.

A PAE cell line stably expressing VEGFR2 and NRP1 was used for signaling assays. This has the advantage that all cells express both receptors at similar concentrations^12^. The phosphorylation of VEGFR2 (PY1175), p38, Erk1/2 and PLCγ was measured 0, 10 and 30 minutes after stimulation with VEGF-A165a. All values were compared to EGFP transduced cells (Fig. 2). Phosphorylation of the receptor at the plasma membrane is the first event after growth factor binding to receptors. This is an intramolecular catalytic reaction that should not be affected by vesicular transport, especially at early time points. Indeed, only 7 of 33 tested RabGAPs led to a change in VEGFR2 phosphorylation (Fig. 2A). If there was a change it was increased phosphorylation, and this increase was only observed 10 minutes after stimulation.

Phosphorylation of VEGFR2 at Y1175 leads to recruitment and activation of PLCγ. As with phosphorylation of VEGFR2, only few RabGAPs changed phosphorylation of PLCγ and if there was a change it was upregulation (Fig. 2B). There was a correlation between the activation of VEGFR2 by phosphorylation at Y1175 and subsequent PLCγ activation: four (TBC1D10A-C and TBCK) of seven RabGAPs that lead to increased phosphorylation of Y1175 also showed increased phosphorylation of PLCγ.

VEGF-induced Erk1/2 signaling is critical for many biological functions such as endothelial cell proliferation, migration, and vessel homeostasis (Simons & Eichmann, 2015). It was shown that the trafficking to early endosomes plays an important role for Erk1/2 signaling. Slow transport gives the PY1175-specific phosphatase PTP1b more time to dephosphorylate VEGFR2, leading to reduced Erk1/2 signaling^15^. We found that seven RabGAPs increase Erk1/2 signaling (Fig. 2C). Not all of them showed increased PLCγ phosphorylation, indicating that there are other pathways acting independently of PLCγ to activate Erk1/2. Interestingly, one RabGAP, TBC1D10B, showed reduced Erk1/2 signaling even though it led to increased phosphorylation of PLCγ. Its nearest orthologue, TBC1D10A, led to increased Erk1/2 and PLCγ phosphorylation.

In contrast to Erk1/2 signaling, p38 MAPK activation is induced by phosphorylation of Y1214 and not Y1175. Full activation of p38 requires NRP1 as coreceptor and Ca2+ influx to cells^25^. The activation of p38 signaling showed less correlation with other measured signaling pathways (Fig. 2D). In contrast to the other assays, we identified nine RabGAPs that reduce signaling. This is probably due to the fact that our VEGF-A165a stimulated PAE cells expressing VEGFR2 and NRP1 are the optimal system for p38 signaling. Only two RabGAPs led to increase p38 phosphorylation.

### Influence of RabGAPs on migration and tube formation of endothelial cells

**Figure 3:**
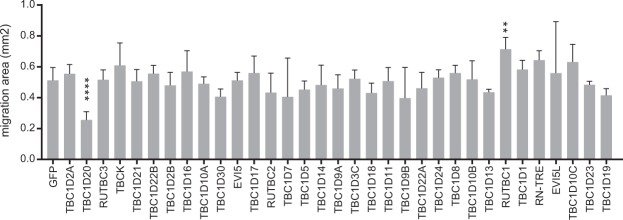
Migration of HUVEC overexpressing RabGAPs. HUVEC were infected with lentivirus expressing EGFP-RabGAPs 40 hours prior to the migration assay. EGFP-transduced HUVEC are shown as control. The assay was stopped after eight hours and the newly covered area was measured. Error bars indicate SEM. **Denotes p < 0.01, ****denotes p < 0.001.

In order to bring our VEGFR2 signaling data into a broader context, we performed migration and tube formation assays with primary endothelial cells (HUVEC). These assays occur in a more complex environment and several growth factor systems are involved such as VEGF, FGF, EGF and IGF-1. To assess the influence of individual RabGAPs on endothelial cell migration, we overexpressed RabGAPs in HUVEC and studied cell migration with a standard assay^26^. We found that most RabGAPs had no effect on migration (Fig. 3). TBC1D20 showed a significant decrease on migration of HUVEC and RUTBC1 had the opposite effect. Both proteins had little influence on VEGFR2 signaling. Expression of TBC1D20 increased Erk1/2 signaling independently of VEGFR2 PY1175 and PLCγ, and RUTBC1 had no effect on signaling, indicating that the observed effects are independent of VEGFR2. Both proteins are not well characterized and no direct link to VEGFR2, angiogenesis or other signaling systems is known. TBC1D20 is a RabGAP effector for Rab1 and Rab2, which govern the trafficking between the endoplasmic reticulum and Golgi^27–29^. Loss of function of TBC1D20 is involved in Warburg Micro syndrome, a disease that is characterized amongst others by cataracts in the lens cause by lack of removal of damaged proteins and organelles from lens fiber cells^30^. RUTBC1 participates in the deactivation of Rab33b, Rab32, Rab38, and Rab9a. Rab33 regulates intra-Golgi trafficking and the formation of autophagosomes. Rab32 mediates the biogenesis of melanosomes. Rab9 controls the trafficking from late endosomes to the TGN^31,32^.

**Figure 4:**
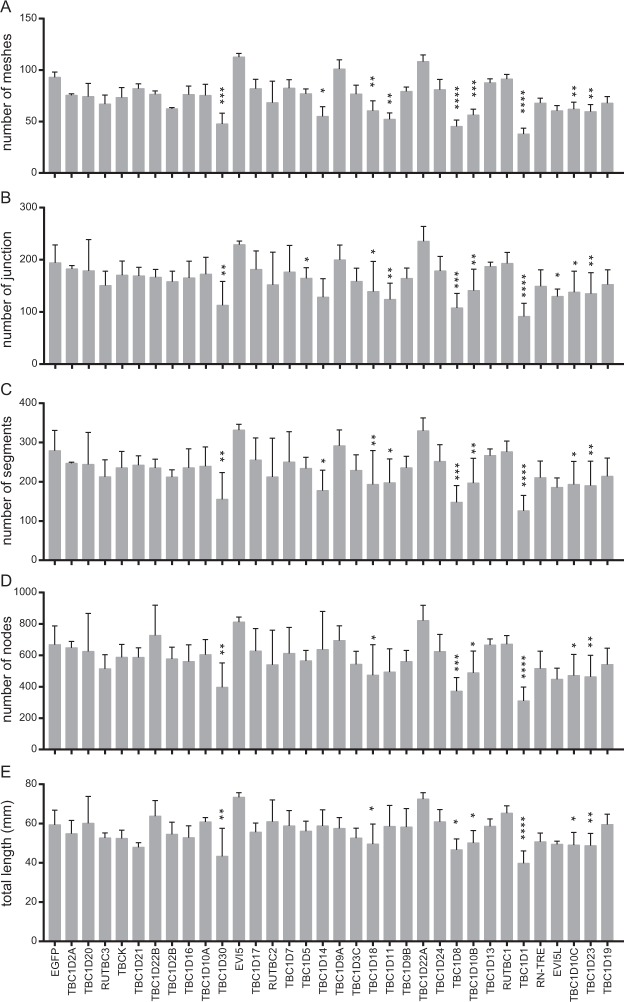
Tube formation of HUVEC overexpressing RabGAPs. HUVEC were transduced with EGFP-RabGAP expressing lentivirus. The different parameters characterizing the tube network are given on the y-axis. *Denotes p < 0.05, **p < 0.01, ***p < 0.005, ****p < 0.001.

Tube formation was first described in 1980 by Folkman and Haudenschild in the context of an *in vitro* angiogenesis assay^33^. Kubota and colleagues found that the formation of capillary-like tubules markedly accelerated when endothelial cells were cultured on a reconstituted gel composed of basement membrane proteins^34^. The endothelial cells retained the capacity to respond to the angiogenic signals and were induced to differentiate and form capillary-like structure^35^. Nowadays tube formation is a widely-used assay to study angiogenic and antiangiogenic factors, to define mechanisms and pathways involved in angiogenesis, and to define endothelial cell populations^36^. We again used lentivirus transduced HUVEC to study the influence of RabGAPs in tube formation. The formation of tubes occurs quickly, beginning one hour after plating cells on the gel matrix substrate. The capillary-like structures were visualized by microscopy six hours after plating, and the number of nodes, junctions, segments, and meshes, as well as the total length of the network, were quantified in ImageJ with “Angiogenesis analyzer”. The EGFP transduced HUVEC function normally when compared to non-treated HUVEC in this angiogenesis assay. Expression of several RabGAPs (TBC1D30, TBC1D18, TBC1D8, TBC1D10B/C, TBC1D1, TBC1D23) induced an impaired tube structure with more disconnections and less well-formed meshes (Fig. 4). TBC1D1 and TBC1D30 are linked to insulin signaling^37,38^. Since insulin is an essential growth factor in the tube formation assay, the observed effect is probably due to interference with this pathway. TBC1D18, TBC1D8, TBC1D23 are not well characterized.

### TBC1D10 subfamily regulates surface expression of VEGFR2 and NRP1

**Figure 5:**
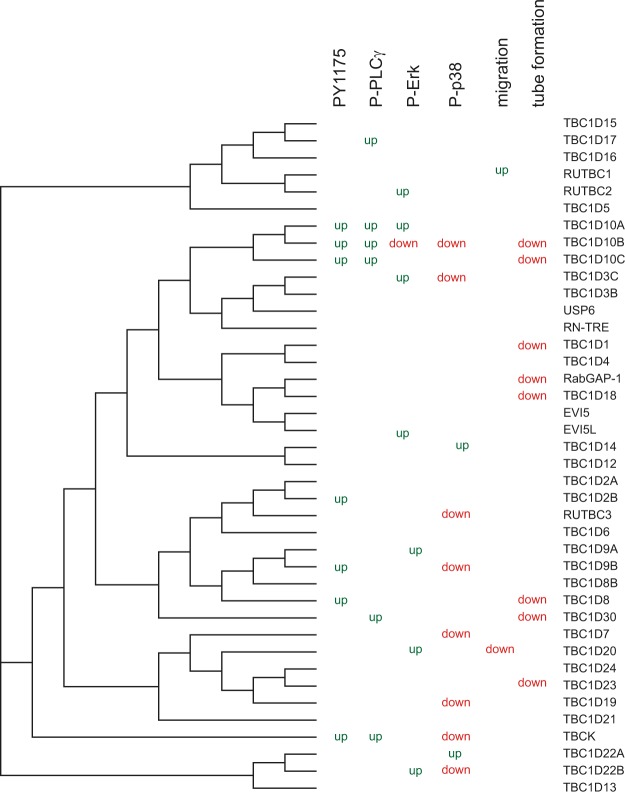
Comparison of obtained result with the phylogenic tree of RabGAPs. The phylogenic tree is based on the conserved domain of Rab GAPs.

**Figure 6:**
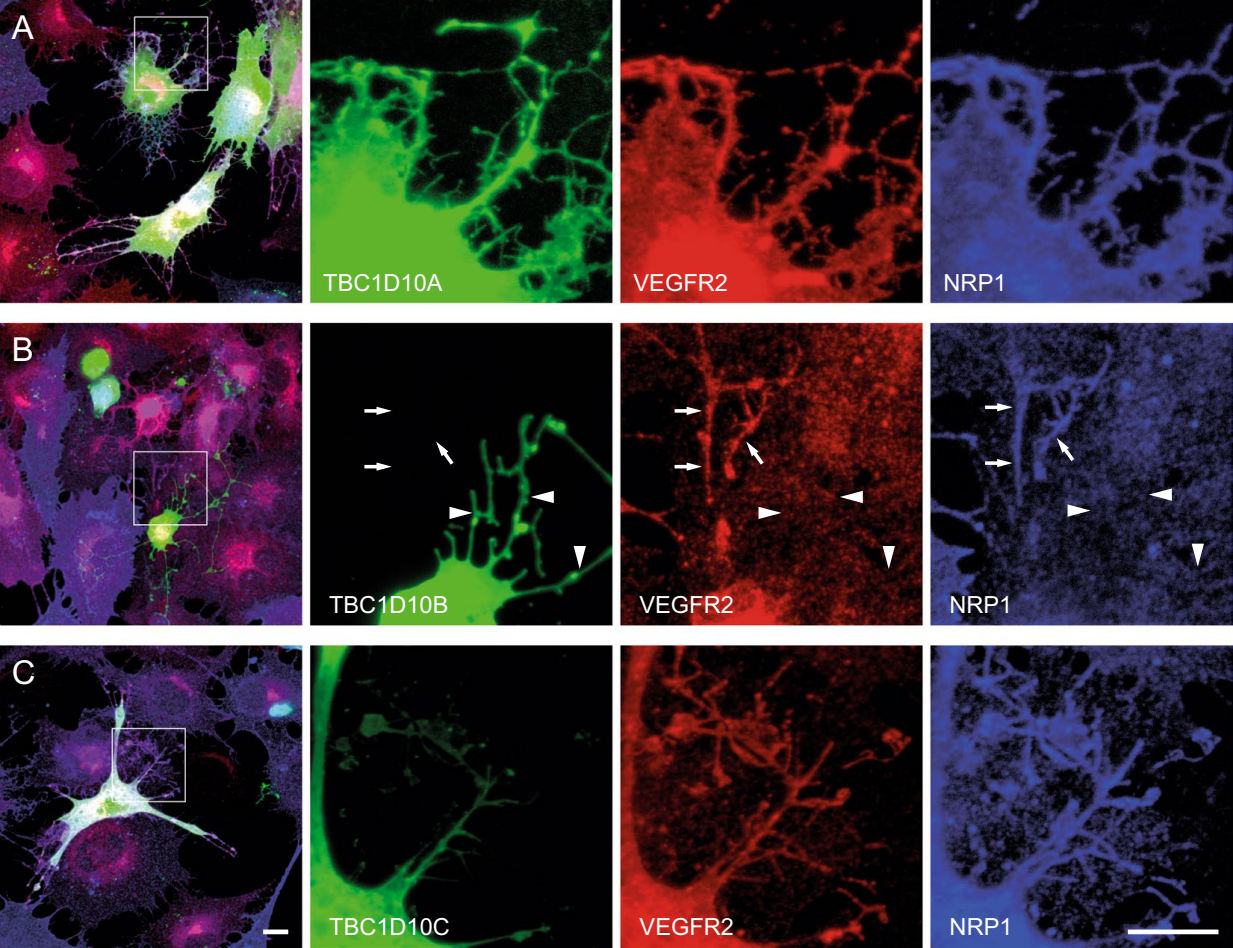
TBC1D10B expression leads to reduced localization of VEGFR2 and NRP1 on filopodia after VEGF-A165a stimulation. (**A**) PAE cells stably expressing VEGFR2 and NRP1 were transiently transfected with TBC1D10A. Cells were fixed 30 minutes after stimulation with VEGF-A165a and immunostained for VEGFR2 and NRP1. VEGFR2 and NRP1 colocalize with TBC1D10A on filopodia. (**B**) Transient transfection of TBC1D10B. TBC1D10B positive filopodia are negative for VEGFR2 and NRP1 (arrowheads). Note that a neighbouring untransfected cell contains VEGFR2 and NRP1 on filopodia (arrows). (**C**) TBC1D10C expressing cells contain VEGFR2 and NRP1 on filopodia. Scale bar: 10 µm.

We interpreted our results in relation to a phylogenic tree of the conserved domain of RabGAPs that was based on the crystal structure of TBC1D15 (Fig. 5 ^39^). The alignment of these domains is given in Supplementary Fig. 1. We found the best link between VEGFR2 signaling and tube formation in the TBC1D10 subfamily consisting of TBC1D10A/ B/ C. Interestingly, TBC1D10A (also known as EPI64), TBC1D10B (EPI64B), and TBC1D10C (carabin) seem to have different effects. TBC1D10B, which downregulated Erk1/2 and p38 signaling, showed a negative effect on tube formation whereas its nearest homologue, TBC1D10A, showed no effect. We then wanted to test how these three RabGAPs influence the localization of VEGFR2 and NRP1 in PAE cells. Under resting conditions these three RabGAPs as well as VEGFR2 and NRP1 are localized at the plasma membrane (Supplementary Fig. 3). Cells were then stimulated for 30 minutes with VEGF A165a. Cells started to form filopodia during this time. We observed that in TBC1D10A expressing cells most of these filopodia were positive for VEGFR2 and NRP1 (78 ± 5%, Fig. 6A). In TBC1D10B expressing cells, filopodia were mostly negative for VEGFR2 and NRP1 (29 ± 6%, Fig. 6B). TBC1D10C led to an intermediate effect (48 ± 8%, Fig. 6C).

**Figure 7:**
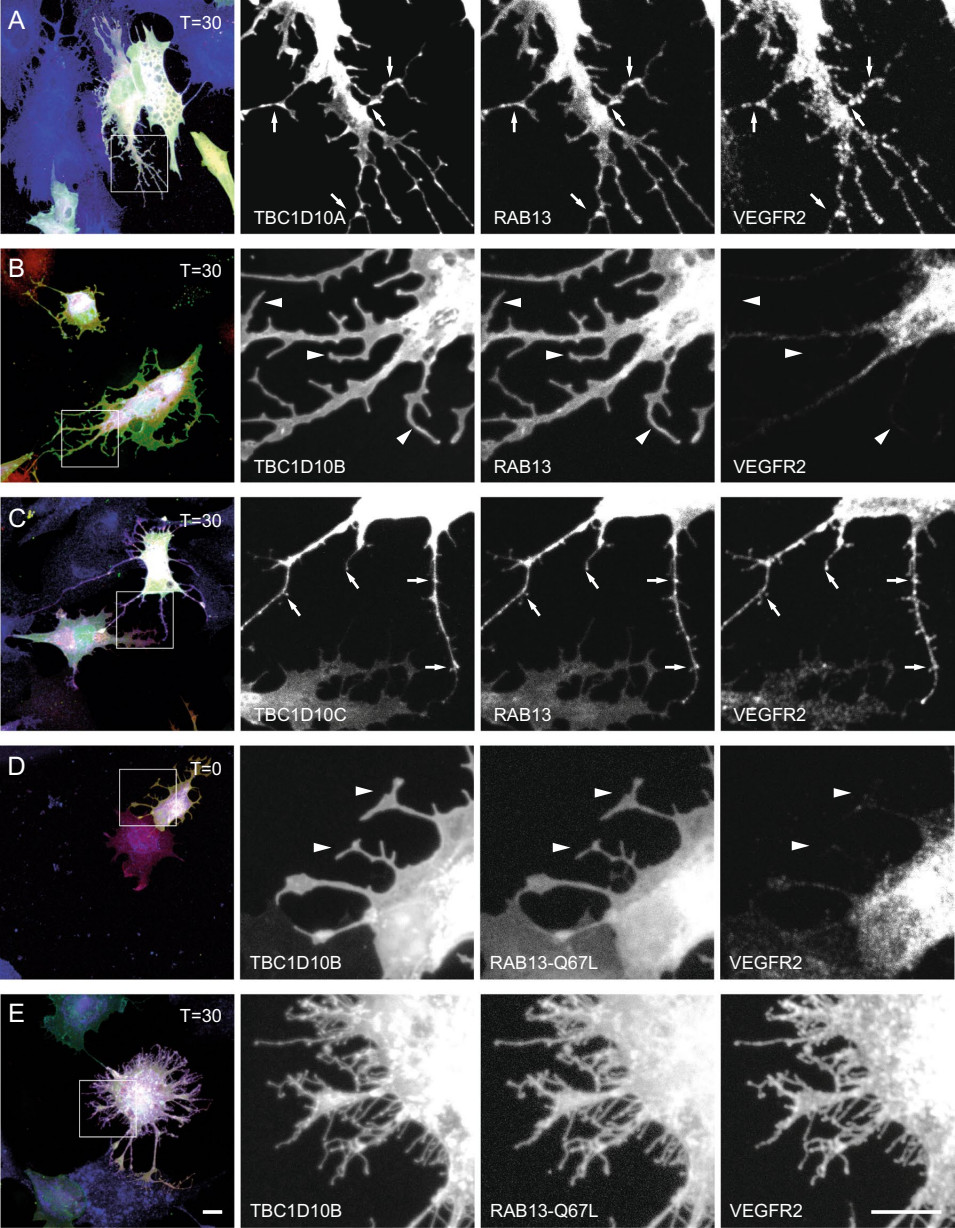
VEGFR2 colocalizes with RAB13 on filopodia in TBC1D10A/C expressing cells after VEGF-A165a stimulation. (**A**) PAE cells stably expressing VEGFR2 and NRP1 were transiently transfected with TBC1D10A and Cherry-RAB13. Cells were fixed 30 minutes after stimulation with VEGF-A165a and immunostained for VEGFR2. VEGFR2 and RAB13 colocalize with TBC1D10A on filopodia (arrows). (**B**) Transient transfection of TBC1D10B and Cherry-RAB13. TBC1D10B positive filopodia are negative for VEGFR2 (arrowheads). TBC1D10B and RAB13 colocalize at the plasma membrane. (**C**) In TBC1D10C and RAB13 expressing cells, TBC1D10C, RAB13, and VEGFR2 colocalize on filopodia (arrows). (**D**) Coexpression of TBC1D10B with a constitutive active form of RAB13. Expression of RAB13-Q67L induces filopodia in unstimulated cells that are negative for VEGFR2 (arrowheads). (**E**) Thirty minutes after stimulation with VEGF A165a, filopodia contain VEGFR2. Scale bar: 10 µm.

Since RAB13 is one of the targets of TBC1D10A and plays a role in VEGFR2 mediated angiogenesis, we coexpressed a Cherry-tagged RAB13^20^. In TBC1D10A/B expressing cells we observed patches containing TBC1D10A/B, VEGFR2 and RAB13 in certain areas of filopodia (Fig. 7A,C). Cells expressing TBC1D10B and RAB13 showed again clearly reduced VEGFR2 expression in filopodia and the expression of these two proteins was more homogenous on the plasma membrane (Fig. 7B). A constitutively active form of Rab13, Rab13-Q67L, was previously shown to induce neurite outgrowth in uninduced PC12 cells^40^. Accordingly, we found an increased number of PAE-VEGFR2-NRP1 cells with filopodia under resting conditions. These filopodia contained VEGFR2 when TBC1D10A/ B were coexpressed (Supplementary Fig. 4) and were negative for VEGFR2 when TBC1D10B was coexpressed (Fig. 7D). After stimulation with VEGF A165a, the filopodia of TBC1D10B expressing cells were positive for VEGFR2 indicating that GTP loaded RAB13 as well as phosphorylated VEGFR2 are important for VEGFR2 transport into filopodia (Fig. 7E). We also used a dominant negative form of RAB13. This mutant, RAB13-T22N, was previously shown to block neurite outgrowth in PC12 cells^40^. In our experiments, this mutant blocked filopodia formation even in stimulated cells (data not shown).

## Discussion

Angiogenesis is a complex process involving attachment, vascular basement membrane degradation, migration, and proliferation of endothelial cells. VEGFR2 is an important player in this context, and here we addressed the influence of RabGAPs on VEGFR2-based signaling, migration, and tube formation. RabGAPs stimulate the slow intrinsic rate of GTP hydrolysis of Rab GTPases. RabGAPs and Rab GTPases organize together with guanine nucleotide exchange factors (GEFs) the directional transport of vesicles and their cargoes (e.g. growth factor receptors) within cells^41^. The use of a RabGAP library to monitor trafficking in endosomal compartment was used before^21,22,42^. VEGFR2 is a good candidate for such an approach, since its trafficking in the endosomal compartment and the consequences for signaling are well documented. Previous studies colocalized VEGFR2 with Rab5, Rab4, Rab11, Rab7, and Rab13^20,43,44^ and it was shown that there exist alternative trafficking routes that have an influence on signaling^12^. As in former studies that used this RabGAP library, we found RabGAPs that specifically influenced effects of VEGFR2. All three members of the TBC1D10 subfamily led to increased phosphorylation of VEGFR2 Y1175 and PLCγ, but only TBC1D10A was associated with increased phosphorylation of Erk1/2 and normal p38 signaling and tube formation, whereas TBC1D10B led to reduced phosphorylation of Erk1/2 and p38, and impaired tube formation. TBC1D10C showed an intermediate behavior promoting upregulation of VEGFR2 and PLCγ phosphorylation, but there was no influence on Erk1/2 or p38 phosphorylation. Opposing effects of proteins of the same family appear on the first view contradictory, but TBC1D10A and TBC1D10B were reported to have different GAP activity on Rab27B. TBC1D10B is a RabGAP for Rab27B and enhanced amylase release from pancreatic acini cells, whereas TBC1D10A did not show this effect^45^. In addition, *in vitro* experiments revealed that these three RabGAPs have different specifities^42^. Structural differences can explain this opposing effect (see Supplementary Fig. 2 for an alignment of the amino acid sequences). TBC1D10B contains at its N-terminus at least one additional domain with 250 amino acids that can add different functions. All three family members attach to the plasma membrane, but TBC1D10A has a PDZ binding motif at its C-terminus that might allow recruitment to a specific receptor complex^46^. For all three TBC1D10 family members the highest GAP activity was reported towards Rab35, and TBC1D10C acts exclusively on Rab35. Mutations in Rab35 that lead to a constitutively active form are associated with cancer development in human. These mutations lead to activation of the PDGFR and increased Akt phosphorylation, and no effect on VEGFR and Erk1/2 phosphorylation was reported^47^. Therefore, it is unlikely that Rab35 mediates the observed effects on signaling and tube formation. TBC1D10A/ B also act on other Rab GTPases such as Rab3A or Rab22A, which are involved in cancer progression, but no difference in substrate specificity was reported^42,48^. Rab13 was previously shown to be involved in VEGFR2 trafficking and is inactivated by TBC1D10A. Rab13 colocalizes with VEGFR2 in polarized endothelial cells at the leading edge in a VEGFR2 PY1175-dependent manner. In zebrafish, Rab13 knockdown leads to a defect in the development of intersegmental vessel, as was observed for other genes involved in angiogenesis^20^. We observe colocalization of TBC1D10A with VEGFR2 and RAB13 on membranes of filopodia suggesting that TBC1D10A and RAB13 cooperate in VEGFR2-mediated angiogenesis. In contrast to this, filopodia of TBC1D10B expressing cells show clearly reduced VEGFR2 and NRP1 expression on the plasma membrane. Since we never observed filopodia lacking VEGFR2 before, we speculate that TBC1D10B promotes withdrawal of VEGFR2/NRP1 from the plasma membrane or prevents its insertion into filopodia. The lack of localization of VEGFR2 on filopodia might also explain the reduced Erk and p38 signaling. Coexpression of a constitutive-active form of RAB13 (RAB13-Q67L) enabled localization of VEGFR2 on filopodia of stimulated cells. This is in line with a previous study that showed that only phosphorylated VEGFR2 can bind to Rab13 in a complex containing Grb2, Actinin-4, and MICAL-L2^20^.

Otherwise we found little overlap between the signaling assay and the migration/ tube formation assays. The migration and tube formation assays are more complex and are done in the presence of several growth factors. It is therefore likely that expression of RabGAPs may affect other signaling systems. Insulin and insulin-like growth factor (IGF) promote tube formation and migration in vascular endothelial cells^49^. FGF-1 (fibroblast growth factor-1) and FGF-2 are other growth factors that promote angiogenesis, and the presence of FGF-1 has been found to increase the tube formation activity of HUVEC^50^. IGF-1 and FGF2 were both present in our assays and their trafficking is influenced by TBC1D1 and TBC1D30^37,38^. This is a possible explanation that these two RabGAPs influence tube formation in a VEGFR2-independent manner.

In summary, we identified the TBC1D10 subfamily of RabGAPs as modulators of VEGFR2 activation, signaling, and trafficking and thus as modulators of endothelial cell tube formation *in vitro*. It was previously shown that these proteins play a role in protein trafficking and vesicle fusion to the plasma membrane. Our study identifies the VEGFR2/NRP1 system as a specific target of the TBC1D10 subfamily and as a new player in angiogenesis.

## Materials and Methods

### Cell culture

Porcine Aortic Endothelial (PAE) cells stably expressing VEGFR2 and Neuropilin-1 (PAE-VEGFR2-NRP1^12^), and HEK293T cells were cultured in DMEM with high glucose (No. 1-26F03-I, BioConcept) supplemented with 10% fetal bovine serum (FBS) and Penicillin/ Streptomycin. Human umbilical vein endothelial cells (HUVEC, Life Technologies) were grown in EGM-2 BulletKit medium (Lonza) containing fetal calf serum, hydrocortisone, hFGF-2, VEGF, R3-IGF-1, ascorbic acid, hEGF, GA-1000 and heparin. Cells were propagated in a humidified atmosphere at 37 °C and 5% CO_2_.

### Lentivirus production

The cloning of a library expressing EGFP-tagged RabGAPs was described previously^21^. The coding regions were transferred into a plasmid suitable for lentivirus production because with the original vectors we did not reach the necessary transfection efficiency in HUVEC and PAE cells (data not shown). The coding regions were amplified with primers GAP_Lenti_For- 5′-ATC CAC CGG TCG CCA CC-3′ and GAP_Lenti_Rev- 5′-ATT GTC GAC GCG GCC GCT-3′. The resulting fragment was cut with AgeI and SalI and ligated into AgeI and SalI cut vector pRRLSIN.cPPT.PGK-GFP.WPRE (gift from Didier Trono (Addgene plasmid #12252)) and then transformed into XL-1Blue E. coli cells. The integrity of all constructs was verified by digesting with several restriction enzymes and sequencing. HEK293T cells were plated at a density of 50% sixteen hours prior to transfection in 175 cm2 cell culture flask. In total 48 μg of DNA mixture with a ratio 3:1:1:1 of the following lentivirus plasmids was prepared: lentiviral gene carrier pRRLSIN.cPPT.PGK-GFP.WPRE with RabGAP insert (Addgene plasmid #12252), pMD2.G (Addgene plasmid #12259, provided by Didier Trono), pMDLg/pRRE and pRSV-Rev^24^. The DNA mixture was transfected with PEI (branched polyethylenimine, Sigma-Aldrich, 1 mg/ml) as transfection reagent at the DNA:PEI ratio of 1:3 in a total volume of 2 ml of OptiMEM (Gibco). The final mixture was incubated for 5–10 mins at room temperature before adding to the cells.

The supernatant containing virus from transfected HEK293T was collected 48–72 hours post-transfection. The cell debris was removed by centrifugation at 200 g for 5 min, and the supernatant was filtered through a 0.45 µm filter. The coarsely-filtered viral supernatant was concentrated by ultracentrifugation (OPTIMA XE Beckman) at 24,000 rpm at 4 °C for 1.5 hours in an SW-28 rotor (Beckman) using Ultra-Clear tubes (Beckman Coulter). The pelleted virus was resuspended in 1/250 of the original volume cold PBS. Virus was stored at −20 °C. For transduction, HUVEC and PAE-VEGFR2-NRP1 cells were seeded in 6-well plates at 50–60% confluency. Since the virus carrying RabGAPs with shorter coding regions was produced more efficiently, less volume of the concentrated virus suspension (100 μl instead of 200 μl) was used to transduce to HUVEC or PAE, vice versa. Thus, comparable transduction efficiency was achieved for all the RabGAP constructs. The cells were used two days after transduction.

### Signaling assays

Transduced PAE-VEGFR2-NRP1 cells were starved in DMEM supplemented with 1% bovine serum albumin (BSA) for 4 hours and then stimulated with 1.5 nM VEGF-A165a for 0, 10, and 30 min. Cells were lysed in lysis buffer (50 mM Tris-HCl, 100 mM NaCl, 1% w/v NP-40, pH 7.5) with protease inhibitors (cOmplete mini, EDTA-free; Roche) and phosphatase inhibitors 20 μM phenylarsine oxide, 1 mM Na_3_VO_4_, 1 mM NaF. Lysates were diluted in 5x SDS-PAGE loading buffer (60 mM Tris-HCl, pH 6.8, 25% glycerol, 2% SDS, 5% β-mercaptoethanol, 0.1% bromophenol blue), heated at 50 °C for 30 minutes, and then separated on 10% SDS-PAGE. Proteins were transferred to PVDF membranes (GE Healthcare), and the membranes were incubated with primary antibodies (dilution 1:1000) followed by secondary alkaline phosphatase-coupled antibodies (1:10000), and developed with Novex AP Chemiluminescent Substrate (Invitrogen). Primary antibodies include: rabbit anti-phospho PLCγ-1 (Cell Signaling; #2821), rabbit anti-PLCγ-1 (Cell Signaling; #2822), rabbit anti-phospho-p38 (Cell Signaling; #9211), rabbit anti-p38 (Sigma-Aldrich; M0800), rabbit anti-VEGFR2 (Cell Signaling, #2479), rabbit anti-phospho-VEGFR2 (pY1175; Cell Signaling; #2478), mouse anti-phospho-p44/42 MAPK (Erk1/2) (Cell Signaling; #9106), rabbit anti-p44/42 MAPK (Erk1/2) (Cell Signaling; #9102), mouse anti-actin (Sigma-Aldrich; A2228), and goat anti-NRP1 (Santa Cruz Biotechnology; sc7239). Secondary antibodies were alkaline phosphatase-coupled goat anti-mouse, anti-goat, and anti-rabbit antibodies (SouthernBiotech). PageRulerTM Plus Prestained Protein Ladder, 10 to 250 kDa (26619, ThermoFisher) was used as protein marker on all gels. Images of immunoblots were captured with an Amersham Imager 600 (GE Healthcare). The blots were analyzed by ImageJ. The raw data were normalized to the signal at 10 minutes of EGFP expressing PAE-VEGFR2-NRP1.

### Statistical analysis

GraphPad Prism software was used for statistical analysis. All data are presented as the mean ± SEM. Statistical significance of differences were determined by using one-way ANOVA followed by Dunnett’s test for multiple comparison of every mean to the control mean. *P* values < 0.01 were considered significant.

### HUVEC migration assay

Migration of RabGAP-infected HUVEC was measured with Culture-Insert 2 Well in µ-Dish 35 mm (Ibidi, Germany). Cells were seeded at a density of 21,000 cells per chamber and allowed to attach overnight. The next day, culture inserts were removed. During the migration assay, cells were cultured under normal conditions, and images were acquired after zero and eight hours with a wide-field fluorescent microscope (Olympus IX81 equipped with Andor iXonEM camera) and analyzed using ImageJ with the plugin Angiogenesis Analyzer.

### HUVEC tube formation assay

15-well μ-Slides (Ibidi, Germany) were coated with 10 μl of 10 mg/ml Geltrex® Matrix (Thermo Fisher) and incubated at 37 °C for 30 minutes to promote solidification. 5000 HUVECs were seeded in growth medium and grown at 37 °C. After 18 h, images were acquired with a wide-field fluorescent microscope (Olympus IX81 equipped with Andor iXonEM camera). Tube formation images were analyzed by using ImageJ plugin Angiogenesis Analyzer (http://image.bio.methods.free.fr/ImageJ/?Angiogenesis-Analyzer-for-ImageJ).

### Immunostainings

PAE cells expressing VEGFR2 and NRP1 were transiently transfected with Lipofectamine 3000 using 1 µg RabGAP and 1 µg carrier DNA or 1 µg RabGAP and 1 µg Cherry-RAB13 DNA according manufacturer recommendations (Thermo Fisher). After 20 hours, cells were starved for three hours with 1% BSA/ DMEM and then stimulated with 1.5 nM VEGF-A165a in 1% BSA/ DMEM for 15 minutes. Cells were fixed with 4% formaldehyde/ PBS, permeabilized with 1% NP40 in PBS for 10 minutes and then incubated with the following antibodies, diluted in PBS: Goat anti-NRP-1 (Santa Cruz Biotechnology, Santa Cruz, USA, sc7239, 1:200), rabbit anti-VEGFR-2 (Cell Signaling, Danvers, USA, #2479, 1:500). Secondary antibodies were labeled with Cy3, or Cy5 (Jackson ImmunoResearch, Suffolk, UK). Samples were analyzed on a Leica SP5 confocal microscope. Maximum projections are shown in figures, levels and gamma settings were adjusted in Adobe Photoshop. All operations were applied to the entire image. For quantification, 50 cells on three independent coverslips were counted.

## Supplementary information

Supplementary Information

## Data Availability

Data and plasmids are available upon request.
