# Supplementary material for: Regulation of VEGFR2 trafficking and signaling by Rab GTPase-activating proteins
Source: Sci Rep. 2019 Sep 16;9:13342. doi: 10.1038/s41598-019-49646-4 (PMC6746989; doi:10.1038/s41598-019-49646-4)
Supplement: Supplementary file 1 — Supplementary Information [file 41598_2019_49646_MOESM1_ESM.pdf]

Supplementary information for

Regulation of VEGFR2 trafficking and signaling by Rab GTPase-activating proteins

Ye Xie[1], Maysam Mansouri[1,2], Aurélien Rizk[1,3], and Philipp Berger[1]*

[1]Paul Scherrer Institute, Laboratory of Nanoscale Biology, CH-5232 Villigen PSI, Switzerland

[2] Present address: ETH Zurich, Department of Biosystems Science and Engineering, CH-4058 Basel, Switzerland

[3] Present address: InterAx Biotech AG, CH-5232 Villigen PSI, Switzerland

Supplementary Figure 1: Alignment RabGAP domains. The RabGAP domain was identified based on the crystal structure of TBC1D15 (Chen et al., 2017). It starts at position 47 (LKI…) and ends at position 488 (…GSE). Conserved amino acids based on this publication are marked in green.

```
                ....|....| ....|....| ....|....| ....|....| ....|....| ....|....| ....|....| ....|....| ....|....| ....|....| ....|....| ....|....|
                    5          15         25         35         45         55         65         75         85         95         105        115
TBC1D15         --------SL RGSDP-STHQ RPPSEMADFL SDAIPG---- ------LKIN QQEEPGFEVI TRID-LGERP VVQRREP--V SLEEWTKNID SEGRILNVDN MKQMIFRGGL SHALRKQAWK
TBC1D17         ------VVSR FLQDPYSTTF SSFSRVTNFF RGALQPQPEG AASDLPPPPD DEPEPGFEVI SCVE-LGPRP TVERGPP--V TEEEWARHVG PEGRLQQVPE LKNRIFSGGL SPSLRREAWK
TBC1D16         ---------- SRESQYKVFH FHHGGLDKLS DVFQQWKYCT EMQLKDQQVA PDKTCMQFSI RRPKLPSSET HPEESMYKRL GVSAWLNHLN ELGQVEEEYK LRKAIFFGGI DVSIRGEVWP
RUTBC1          ---------- RLIHRDSTIS NDVFISVDDL EPPEPQDPED SRPKP----E QEAGPGTPGT AVVE-QQHSV EFDSPDSGLP SSRNYSVASG IQSSLDEGQS VGFEEEDGGG EEGSSGPGPA
RUTBC2          ---------- NFSSGLSEHS EPSLSTEDSV LDAQRNTPTV LRPRDGSVDD RQSSEATTSQ DEAP-REELA VQDSLESDLL ANESMDEFMS ITGSLD---- MALPEKDDVV MEGWRSSETE
TBC1D5          --MYHSLSET RHPLQPEEQE VGIDPLSSYS NKSGGDSNKN GRRTSSTLDS EGTFNSYRKE WEELFVNNNY LATIRQKGIN GQLRSSRFRS ICWKLFLCVL PQDKSQWISR IEELRAWYSN
TBC1D10A        ---------- ---------- ---------S LGSDSE---A NG-FAERRID KFGFIVGSQG AEGALEEVP- LEVLRQR--- ESKWLDMLNN WDKWMAKKHK KIRLRCQKGI PPSLRGRAWQ
TBC1D10B        ---------- ---------- ---------S MGSDSE---I NG-LALRKTD KYGFLGGSQY SGSLESSIP- VDVARQR--- ELKWLDMFSN WDKWLSRRFQ KVKLRCRKGI PSSLRAKAWQ
TBC1D10C        ---------- ---------- ---------S LGSDSE---L SGPGPYRQAD RYGFIGGSS- AEPGPGHPP- ADLIRQR--- EMKWVEMTSH WEKTMSRRYK KVKMQCRKGI PSALRARCWP
TBC1D3C         ---MDVVEVA GSWWAQERED IIMKYEKGHR AGLPEDKGPK PFRSYNNNVD HLGIVHETEL PPLTAREAKQ IRREISR--- KSKWVDMLGD WEKYKSS--R KLIDRAYKGM PMNIRGPMWS
TBC1D3B         ---MDVVEVA GSWWAQERED IIMKYEKGHR AGLPEDKGPK PFRSYNNNVD HLGIVHETEL PPLTAREAKQ IRREISR--- KSKWVDMLGD WEKYKSS--R KLIDRAYKGM PMNIRGPMWS
USP6            ---MDMVENA DSLQAQERKD ILMKYDKGHR AGLPEDKGPE PVG-INSSID RFGILHETEL PPVTAREAKK IRREMTR--- TSKWMEMLGE WETYKHS--S KLIDRVYKGI PMNIRGPVWS
RN-TRE          ---MNSDQDV ALKLAQERAE IVAKYDRG-R EGAEIEPWED ADYLVYKVTD RFGFLHEEEL PDHNVAVERQ KHLEIER--- TTKWLKMLKG WEKYKNT--E KFHRRIYKGI PLQLRGEVWA
TBC1D1          ---------- --GPPPEEKK RTSRELRELW QKAILQQILL LRMEKENQKL QASENDLLNK RLKLDYEEIT PCLKEVT--- TVWEKMLSTP GRSKIKFDME KMHSAVGQGV PRHHRGEIWK
TBC1D4          -EPLVVFLSG EDDPEKIEER KKSKELRSLW RKAIHQQILL LRMEKENQKL EASRDELQSR KVKLDYEEVG ACQKEVL--- ITWDKKLLN- CRAKIRCDME DIHTLLKEGV PKSRRGEIWQ
RabGAP-1        -------VCL ESESERERRK TTASPSVRLP QSGSQSSVIP -------SPP EDDEEEDNDE PLLSGSGDVS KECAEKI--- LETWGELLSK WHLNLNVRPK QLSSLVRNGV PEALRGEVWQ
TBC1D18         ---------S EGKGHTNAGD AIYEVVSLQR ESDKEEPVTP TSGGGPMSPQ DDEAEEESDN ELSSGTGDVS KDCPEKI--- LYSWGELLGK WHSNLGARPK GLSTLVKSGV PEALRAEVWQ
EVI5            ---------T PALSPSSPSQ LSPDDLELLA KLEEQNRLLE TDSKSLRSVN GSRRNSGSSL VSSSSASSNL SHLEEDS--- WILWGRIVNE WEDVRKKKEK QVKELVHKGI PHHFRAIVWQ
EVI5L           ---------- ----TSDSEN LSPDELELLA KLEEQNRLLE ADSKSMRSMN GSRRNSGSSL VSSSSASSNL SHLEEDT--- WILWGRIANE WEEWRRRKEK LLKELIRKGI PHHFRAIVWQ
TBC1D14         -PKQNVRKNL DFEPLSTTAL ILEDRPANLP AKPAEEAQKH RQQYEEMVVQ AKKRELKEAQ RRKKQLEERC RVEESIGNAV LTWNNEILPN WETMWCS--R KVRDLWWQGI PPSVRGKVWS
TBC1D12         --QSSRRKNF EFEPLSTTAL ILEDRPSNLP AKSVEEALRH RQEYDEMVAE AKKREIKEAH RKRKIMKERF KQEENIASAM VIWINEILPN WEVMRST--R RVRELWWQGL PPSVRGKVWS
TBC1D2A         ---------- --ALQWEAGE ASSDS-IELS PISKYDEYGF LTVPDYEVED LKLLAKIQAL ES-RSHHLLG LEAVDRP--- LRER--WAAL G---DLVPSA ELKQLLRAGV PREHRPRVWR
TBC1D2B         ---------- --ALQVESQE QPEQAFVKPH LVSEYDIYGF RTVPEDDEEE -KLVAKVRAL DL-KTLYLTE NQEVSTG--- VKWENYFAST VN-REMMCSP ELKNLIRAGI PHEHRSKVWK
RUTBC3          ---------- --SIWPQEIL AKYTQKEESA EQPEFYYDEF GFRVYKEEGD EPGSSLLANS PLMEDAPQRL RWQAHLEFTH NHDVGDLTWD KIAVSLPRSE KLRSLVLAGI PHGMRPQLWM
TBC1D6          ---------- ---------- ------MQPA ERSRVPRIDP YGFERPEDFD DAAYEKFFSS YLVTLTRRAI KWSRLLQ--- ---------- --GGGVPRSR TVKRYVRKGV PLEHRARVWM
TBC1D9A         ---------- ---YSRPSSL VSSSPQRSTS SDADGERQFN LNGNSVPTAT QTLMTMYRRR SP-EEFNPKL AKEFLKE--- QAWKIHFAEY GQGICMYRTE KTRELVLKGI PESMRGELWL
TBC1D9B         ---------- ----SSPAPQ EGSEQPASPA SPLSSRQSF- -CAQEAPTAS QGLLKLFQKN SPMEDLGAKG AKEKMKE--- ESWHIHFFEY GRGVCMYRTA KTRALVLKGI PESLRGELWL
TBC1D8B         ---LRCGAAS TQYHDISTEL AISSESTEPS DNFEVQSLTS QRECSKTVNT EALMTVFHPQ NL-ETLNSKM LKEKMKE--- QSWKILFAEC GRGVSMFRTK KTRDLVVRGI PETLRGELWM
TBC1D8          ----SADDDM ASLVFHSTSM CSDHRFGDLE MMSSQNSEES EKEKSPLMHP DALVTAFQQS GS-QSPDSRM SREQIKI--- SLWNDHFVEY GRTVCMFRTE KIRKLVAMGI PESLRGRLWL
TBC1D30         ---------- -MRQDKLTGS LRRGGRCLKR QGGGVGTILS NVLKKRSCIS RTAPRLLCTL EPGVDTKLKF TLEPSLG--- ---------Q NGFQQWYDAL KAVARLSTGI PKEWRRKVWL
TBC1D7          ---------- ---------- -MTEDSQRNF RSVYYEKVGF RGVEEKKSLE ILLKDDRLDT EKLCTFSQRF PLPSMYR--- ---------A LVWKVLLGIL PPHHESHAKV
TBC1D20         ---------- -MALRSAQGD GPTSGHWDGG AEKADFNAKR KKKVAEIHQA LNSDPTDVAA LRRMAISEGG LLTDEIRRKV WPKLLNVNAN DPPPISGKNL RQMSKDYQQV LLDVRRSLRR
TBC1D24         --VTPDASVY SDIVGKIVGK HSSSCLPLPE FVDNTQVPSY CLNARGEGAV RKILLCLANQ FPDISFCPAL PAVVALLLHY SIDEAECFEK ACRILACNDP GRRLIDQSFL AFESSCMTFG
TBC1D23         ----LFRLLI QYHEPELCSY LDTKKITPDS YALNWLGSLF ACYCSTEVTQ AIWDGYLQQA DPFFIYFLML IILVNAKEVI LTQESDSKEE VIKFLENTPS SLNIEDIEDL FSLAQYYCSK
TBC1D19         PEHLKEPLVY MRKAQGSWEK RILKSLNSMC TELSIPLARK RPVGEQKELL NKWNEMGTDE PDLSLFRPVY APKDFLEVLI NLRNPNYENG DSLSFRTHLG LIQVPLKVKD IPELKECFVE
TBC1D21         ---------- --------MT TLSPENSLSA RQSASFILVK RKPPIDKTEW DSFFDESGHL AKSRDFICVN ILERGLHPFV RTEAWKFLTG YFSWQSSQDE RLTVDSMRRK NYKALCQMYE
TBCK            -------SFG QGRDRSSLLD DTTVTLSLCQ LRNRLKDVGG EAFYPLLEDD QSNLPHSNSN NELSAAATLP LIIREKDTEY QLNRIILFDR LLKAYPYKKN QIWKEARVDI PPLMRGLTWA
TBC1D22A        ---------P RPEAEPPSPP SGDLRLVKSV SESHTSCPAE SASDAAPLQR SQSLPHSATV TLGG-TSDPS TLSSSALSER EASRLDKFKQ LLAGPNTDLE ELRRLSWSGI PKPVRPMTWK
TBC1D22B        ---------- RSQSTTSDVP AN-YKVIKSS SDAQLSRNSS DTCLRNPLHK QQSLPLRPII PLVARISDQN ASGAPPMTVR EKTRLEKFRQ LLSSQNTDLD ELRKCSWPGV PREVRPITWR
TBC1D13         -------IPC EGGLRCLCWK ILLNYLPLER ASWTSILAKQ RELYAQFLRE MIIQPGIAKA NMGVSREDVT FEDHPLNPNP DSRWNTYFKD NEVLLQIDKD VRRLCPDISF FQRATDYPCL
```

```
               ....|....| ....|....| ....|....| ....|....| ....|....| ....|....| ....|....| ....|....| ....|....| ....|....| ....|....| ....|....|
                     125        135        145        155        165        175        185        195        205        215        225        235
TBC1D15        FLLGYFPWDS TKEERTQLQK QKTDEYFRMK LQWKSISQ-- -----EQEKR NSRLRDYRSL IEKDVNRTDR TNKFYEGQDN -PGLILLHDI LMTYCMYDFD LG-----YVQ GMSDLLSPLL
TBC1D17        FLLGYLSWEG TAEEHKAHIR KKTDEYFRMK LQWKSVSP-- -----EQERR NSLLHGYRSL IERDVSRTDR TNKFYEGPEN -PGLGLLNDI LLTYCMYHFD LG-----YVQ GMSDLLSPIL
TBC1D16        FLLRYYSHES TSEEREALRL QKRKEYSEIQ QKRLSMTP-- -------EEH RAFWRNVQFT VDKDVVRTDR NNQFFRGEDN -PNVESMRRI LLNYAVYNPA VG-----YSQ GMSDLVAPIL
RUTBC1         AHTLREPQDP SQEKPQAGEL EAGEELAAVC AAA------- -----YTIEL LDTVALNLHR IDKDVQRCDR NYWYFTPPN- ---LERLRDV MCSYVWEHLD VG-----YVQ GMCDLLAPLL
RUTBC2         KHGQADSEDN LSEEPEMESL FPALASLAVT TSANEVSPVS SSGVTYSPEL LDLYTVNLHR IEKDVQRCDR NYWYFTPAN- ---LEKLRNI MCSYIWQHIE IG-----YVQ GMCDLLAPLL
TBC1D5         IKEIHITNP- --------RK VVGQQDLMIN NPLSQDEG-- -----SLWNK FFQDKELRSM IEQDVKRTFP EMQFFQQENV ---RKILTDV LFCYARENEQ LL-----YKQ GMHELLAPIV
TBC1D10A       YLSG------ ---------- -GKVKLQQNP GK----FD-- -----ELDMS -PGDPKWLDV IERDLHRQFP FHEMFVSRGG -HGQQDLFRV LKAYTLYRPE EG-----YCQ AQAPIAAVLL
TBC1D10B       YLSN------ ---------- -SKELLEQNP GK----FE-- -----ELERA -PGDPKWLDV IEKDLHRQFP FHEMFAARGG -HGQQDLYRI LKAYTIYRPD EG-----YCQ AQAPVAAVLL
TBC1D10C       LLCG------ ---------- -AHVCQKNSP GT----YQ-- -----ELAEA -PGDPQWMET IGRDLHRQFP LHEMFVSPQG -HGQQGLLQV LKAYTLYRPE QG-----YCQ AQGPVAAVLL
TBC1D3C        VLLN------ ---------- -TEEMKLKNP GR----YQ-- -----IMKEK GKRSSEHIQR IDRDVSGTLR KHIFFRDRYG -TKQRELLHI LLAYEEYNPE VG-----YCR DLSHIAALFL
TBC1D3B        VLLN------ ---------- -IEEMKLKNP GR----YQ-- -----IMKEK GKRSSEHIQR IDRDISGTLR KHMFFRDRYG -TKQRELLHI LLAYEEYNPE VG-----YCR DLSHIAALFL
USP6           VLLN------ ---------- -IQEIKLKNP GR----YQ-- -----IMKER GKRSSEHIHH IDLDVRTTLR NHVFFRDRYG -AKQRELFYI LLAYSEYNPE VG-----YCR DLSHITALFL
RN-TRE         LLLE------ ---------- -IPKMKEETR DL----YS-- -----KLKHR ARGCPSDIRQ IDLDVNRTFR DHIMFRDRYG -VKQQSLFHV LAAYSIYNTE VG-----YCQ GMSQITALLL
TBC1D1         FLAE------ ---------- -QFHLKHQFP SKQQPKDV-- -----PYKEL LKQLTSQQHA ILIDLGRTFP THPYFSAQLG -AGQLSLYNI LKAYSLLDQE VG-----YCQ GLSFVAGILL
TBC1D4         FLAL------ ---------- -QYRLRHRLP NKQQPPDI-- -----SYKEL LKQLTAQQHA ILVDLGRTFP THPYFSVQLG -PGQLSLFNL LKAYSLLDKE VG-----YCQ GISFVAGVLL
RabGAP-1       LLAG------ ---------- -CHNNDHLVE K--------- ------YRIL ITKESPQDSA ITRDINRTFP AHDYFKDTGG -DGQDSLYKI CKAYSVYDEE IG-----YCQ GQSFLAAVLL
TBC1D18        LLAG------ ---------- -CHDNQAMLD R--------- ------YRIL ITKDSAQESV ITRDIHRTFP AHDYFKDTGG -DGQESLYKI CKAYSVYDED IG-----YCQ GQSFLAAVLL
EVI5           LLCS------ ---------- -AQS-MPIKD Q--------- ------YSEL LKMTSPCEKL IRRDIARTYP EHNFFKEKDS -LGQEVLFNV MKAYSLVDRE VG-----YCQ GSAFIVGLLL
EVI5L          LLCS------ ---------- -ATD-MPVKN Q--------- ------YSEL LKMSSPCEKL IRRDIARTYP EHEFFKGQDS -LGQEVLFNV MKAYSLVDRE VG-----YCQ GSAFIVGLLL
TBC1D14        LAIGNELNIT HELFDICLAR AKERWRSLST GGSEVENE-- -----DAGFS AADREASLEL IKLDISRTFP NLCIFQ-QGG -PYHDMLHSI LGAYTCYRPD VG-----YVQ GMSFIAAVLI
TBC1D12        LAVGNELNIT PELYEIFLSR AKERWKSFSE TSS--END-- -----TEGVS VADREASLEL IKLDISRTFP SLYIFQ-KGG -PYHDVLHSI LGAYTCYRPD VG-----YVQ GMSFIAAVLI
TBC1D2A        WLVH------ ---------- -LRVQHLHT- --PGCYQE-- -----LLSRG QAREHPAARQ IELDLNRTFP NNKHFTCPTS -SFPDKLRRV LLAFSWQNPT IG-----YCQ GLNRLAAIAL
TBC1D2B        WCVD------ ---------- -RHTRKFKDN TEPGHFQT-- -----LLQKA LEKQNPASKQ IELDLLRTLP NNKHYSCPTS -EGIQKLRNV LLAFSWRNPD IG-----YCQ GLNRLVAVAL
RUTBC3         RLSG------ ---------- -ALQKKRNSE LSYR------ ----EIVKNS SNDETIAAKQ IEKDLLRTMP SNACFASMGS -IGVPRLRRV LRALAWLYPE IG-----YCQ GTGMVAACLL
TBC1D6         VLSG------ ---------- -AQAQMDQNP GYY------- -----HQLLQ GERNPRLEDA IRTDLNRTFP DNVKFRKTTD PCLQRTLYNV LLAYGHHNQG VG-----YCQ GMNFIAGYLI
TBC1D9A        LLSG------ ---------- -AINEKATH- --PGYYED-- -----LVEKS MGKYNLATEE IERDLHRSLP --EHPAFQNE -MGIAALRRV LTAYAFRNPN IG-----YCQ AMNIVTSVLL
TBC1D9B        LFSG------ ---------- -AWNEMVTH- --PGYYAE-- -----LVEKS TGKYSLATEE IERDLHRSMP --EHPAFQNE -LGIAALRRV LTAYAFRNPT IG-----YCQ AMNIVTSVLL
TBC1D8B        LFSG------ ---------- -AVNDMATN- --PDYYTE-- -----VVEQS LGTCNLATEE IERDLRRSLP --EHPAFQSD -TGISALRRV LTAYAYRNPK IG-----YCQ AMNILTSVLL
TBC1D8         LFSD------ ---------- -AVTDLASH- --PGYYGN-- -----LVEES LGKCCLVTEE IERDLHRSLP --EHPAFQNE -TGIAALRRV LTAYAHRNPK IG-----YCQ SMNILTSVLL
TBC1D30        TLAD------ ---------- -HYLHSIAID WDKTMRFT-- -----FNERS NPDDDSMGIQ IVKDLHRTGC SSYCGQEAEQ --DRVVLKRV LLAYARWNKT VG-----YCQ GFNILAALIL
TBC1D7         MMYR------ ---------- ---------- ---------- --------KE QYLDVLHALK VVRFVSDATP QAEVYLRMYQ --LESGKLPR SPSFPLEPDD EV-----FLA IAKAMEEMVE
TBC1D20        FPPG------ ---------- MPEEQREGLQ EELIDIILL- ----ILERNP QLHYYQGYHD IVVTFLLVVG ERLATSLVEK LSTHHLRDFM DPTMDNTKHI LN-----YLM PIIDQVNPEL
TBC1D24        DLVN------ KYCQAAHKLM VAVSEDVLQV YADWQRWLFG ELPLCYFARV FDVFLVEGYK VLYRVALAIL KFFHKVRAGQ PLESDSVKQD IRTFVRDIAK TV-----SPE KLLEKAFAIR
TBC1D23        TPAS------ ---------- FRKDNHHLFG STLLGIKDDD -----ADLSQ ALCLAISVSE ILQANQLQGE GVRFFVVDCR PAEQYNAGHL STAFHLDSDL MLQNPSEFAQ SVKSLLEAQK
TBC1D19        LGLNIGQLG- IDDSTQVPPE LFENEHVRIG QKVLAEQDSA AAQQYIRQGS PTALRAELWA LILNISSQPE DVLYYEQLKT NVIQHDLLVD SLIYKDVKLT ASND--DYYF VFEDYLYQVL
TBC1D21        KIQP------ ---------- ---------- ---------- -----LLENL HRNFTETRNN IARDIQKIYD KDPLGNVLID ---KKRLEKI LLLSYVCNTQ AE-----YQQ GFHEMMMLFQ
TBCK           ALLG------ ---------- -VEGAIHAK- ---------- -----YDAID KDTPIPTDRQ IEVDIPRCHQ YDELLSSPEG ---HAKFRRV LKAWVVSHPD LV-----YWQ GLDSLCAPFL
TBC1D22A       LLSGYLPAN- -VDRRPATLQ RKQKEYFAFI EHY------- -----YDSRN DEVHQDTYRQ IHIDIPRMSP -EALILQPK- --VTEIFERI LFIWAIRHPA SG-----YVQ GINDLVTPFF
TBC1D22B       LLSGYLPAN- -TERRKLTLQ RKREEYFGFI EQY------- -----YDSRN EEHHQDTYRQ IHIDIPRTNP LIPLFQQPL- --VQEIFERI LFIWAIRHPA SG-----YVQ GINDLVTPFF
TBC1D13        LILD------ ---------- -PQNEFETLR KRVEQTTLKS ----QTVARN RSGVTNMSSP HKNSVPSSLN EYEVLPNGCE -AHWEVVERI LFIYAKLNPG IA-----YVQ GMNEIVGPLY
```

```
              ....|....| ....|....| ....|....| ....|....| ....|....| ....|....| ....|....| ....|....| ....|....| ....|....| ....|....| ....|....|
                    245        255        265        275        285        295        305        315        325        335        345        355
TBC1D15       YVME------ ---------- ---------- -NEVDAFWCF ASYMDQ---- MHQNFEEQMQ GMKTQLIQL- ---------- ---------- -STLLRLLDS GFCSYLESQ- ----------
TBC1D17       YVIQ------ ---------- ---------- -NEVDAFWCF CGFMEL---- VQGNFEESQE TMKRQLGRL- ---------- ---------- -LLLLRVLDP LLCDFLDSQ- ----------
TBC1D16       AEVL------ ---------- ---------- -DESDTFWCF VGLMQN---- TIFVSSPRDE DMEKQLLYL- ---------- ---------- -RELLRLTHV RFYQHLVSLG ----------
RUTBC1        VTLD------ ---------- ---------- -NDQLAYSCF SHLMKR---- MSQNFPNG-G AMDTHFANM- ---------- ---------- -RSLIQILDS ELFELMHQNG ----------
RUTBC2        VILD------ ---------- ---------- -DEALAFSCF TELMKR---- MNQNFPHG-G AMDTHFANM- ---------- ---------- -RSLIQILDS ELFELMHQNG ----------
TBC1D5        FVLHCDHQAF LHASESAQPS EEMKTVLNPE YLEHDAYAVF SQLMETAEPW FSTFEHDGQK GKETLMTPIP FARPQDLGPT IAIVTKVNQI QDHLLKKHDI ELYMHLNRL- ----------
TBC1D10A      MHMP------ ---------- ---------- -AEQ-AFWCL VQICEK---Y LPGYYSEKLE AIQLDGEIL- ---------- ---------- -FSLLQKVSP VAHKHLSRQ- ----------
TBC1D10B      MHMP------ ---------- ---------- -AEQ-AFWCL VQICDK---Y LPGYYSAGLE AIQLDGEIF- ---------- ---------- -FALLRRASP LAHRHLRRQ- ----------
TBC1D10C      MHLP------ ---------- ---------- -PEE-AFWCL VQICEV---Y LPGYYGPHME AVRLDAEVF- ---------- ---------- -MALLRRLLP HVHKHLQQV- ----------
TBC1D3C       LYLP------ ---------- ---------- -EED-AFWAL VQLLASERHS LQGFHSPNGG TVQGLQDQQ- ---------- ---------- -EHVVATSQP KTMGHQDKK- ----------
TBC1D3B       LYLP------ ---------- ---------- -EED-AFWAL VQLLASERHS LQGFHSPNGG TVQGLQDQQ- ---------- ---------- -EHVVATSQS KTMGHQDKK- ----------
USP6          LYLP------ ---------- ---------- -EED-AFWAL VQLLASERHS LPGFHSPNGG TVQGLQDQQ- ---------- ---------- -EHVVPKSQP KTMWHQDKE- ----------
RN-TRE        MYMN------ ---------- ---------- -EED-AFWAL VKLFSGPKHA MHGFFVQGFP KLLRFQEHH- ---------- ---------- -EKILNKFLS KLKQHLDSQ- ----------
TBC1D1        LHMS------ ---------- ---------- -EEE-AFKML KFLMFDMG-- LRKQYRPDMI ILQIQMYQL- ---------- ---------- -SRLLHDYHR DLYNHLEEH- ----------
TBC1D4        LHMS------ ---------- ---------- -EEQ-AFEML KFLMYDLG-- FRKQYRPDMM SLQIQMYQL- ---------- ---------- -SRLLHDYHR DLYNHLEEN- ----------
RabGAP-1      LHMP------ ---------- ---------- -EEQ-AFSVL VKIMFDYG-- LRELFKQNFE DLHCKFYQL- ---------- ---------- -ERLMQEYIP DLYNHFLDI- ----------
TBC1D18       LHMP------ ---------- ---------- -EEQ-AFCVL VKIMYDYG-- LRDLYRNNFE DLHCKFYQL- ---------- ---------- -ERLMQEQLP DLHSHFSDL- ----------
EVI5          MQMP------ ---------- ---------- -EEE-AFCVF VKLMQDYR-- LRELFKPSMA ELGLCMYQF- ---------- ---------- -ECMIQEHLP ELFVHFQSQ- ----------
EVI5L         MQMP------ ---------- ---------- -EEE-AFCVF VRLMQEYR-- LRELFKPSMA ELGLCIYQF- ---------- ---------- -EYMLQEQLP DLNTHFRSQ- ----------
TBC1D14       LNLD------ ---------- ---------- -TAD-AFIAF SNLLNKP--C QMAFFRVDHG LMLTYFAAF- ---------- ---------- -EVFFEENLP KLFAHFKKN- ----------
TBC1D12       LNLE------ ---------- ---------- -EAD-AFIAF ANLLNKP--C QLAFFRVDHS MMLKYFATF- ---------- ---------- -EVFFEENLS KLFLHFKSY- ----------
TBC1D2A       LVLE------ ---------- ---------- -EEESAFWCL VAIVET--IM PADYYCNTLT ASQVDQRVL- ---------- ---------- -QDLLSEKLP RLMAHLGQH- ----------
TBC1D2B       LYLE------ ---------- ---------- -QED-AFWCL VTIVEV--FM PRDYYTKTLL GSQVDQRVF- ---------- ---------- -RDLMSEKLP RLHGHFEQY- ----------
RUTBC3        LFLE------ ---------- ---------- --EEDAFWMM SAIIED--LL PASYFSTTLL GVQTDQRVL- ---------- ---------- -RHLIVQYLP RLDKLLQEH- ----------
TBC1D6        LITN------ ---------- ---------- -NEEESFWLL DALVGR---I LPDYYSPAML GLKTDQEVL- ---------- ---------- -GELVRAKLP AVGALMERLG ----------
TBC1D9A       LYAK------ ---------- ---------- -EEE-AFWLL VALCER--ML P-DYYNTRVV GALVDQGVF- ---------- ---------- -EELARDYVP QLYDCMQDL- ----------
TBC1D9B       LYGS------ ---------- ---------- -EEE-AFWLL VALCER--ML P-DYYNTRVV GALVDQGIF- ---------- ---------- -EELTRDFLP QLSEKMQDL- ----------
TBC1D8B       LYAK------ ---------- ---------- -EEE-AFWLL VAVCER--ML P-DYFNRRII GALVDQAVF- ---------- ---------- -EELIRDHLP QLTEHMTDM- ----------
TBC1D8        LYTK------ ---------- ---------- -EEE-AFWLL VAVCER--ML P-DYFNHRVI GAQVDQSVF- ---------- ---------- -EELIKGHLP ELAEHMNDL- ----------
TBC1D30       EVME------ ---------- ---------- GNEGDALKIM IYLIDK--VL PESYFVNNLR ALSVDMAVF- ---------- ---------- -RDLLRMKLP ELSQHLDTLQ RTANKESGGG
TBC1D7        DSVD------ ---------- ---------- -----CYWIT RRFVNQ---- LNTKYRDSLP QLPKAFEQY- ---------- ---------- ----LNLEDG RLLTHLRMCS ----------
TBC1D20       HDFMQS---- ---------- ---------- -AEVGTIFAL SWLITWFG-H VLSDFRHVVR LYDFFLACH- ---------- ---------- --PLMPIYFA AVIVLYREQE ----------
TBC1D24       LFSR------ ---------- ---------- --KEIQLLQM ANEKALKQKG ITVKQKRQFV HLAVHAENFR ---------- ---------- SEIVSVREMR DIWSWVPER- ----------
TBC1D23       QSIESG---- ---------- ---------- SIAGGEHLCF MGSGREEEDM YMNMVLAHFL QKNKEYVSIA SG-------- ---------- GFMALQQHLA DINVDGPENG YG--------
TBC1D19       LCFSR----- ---------- ---------- DTSVLSHFAF NSASPPKSYI RGKLGLEEYA VFYPPNGVIP ---------- ---------- -FHGFSMYVA PLCFLYHEPS K---------
TBC1D21       LMVE------ ---------- ---------- -HDHETFWLF QFFLQK---- -TEHSCVINI GVAKNLDML- ---------- ---------- -STLITFLDP VFAEHLKGK- ----------
TBCK          YLNFN----- ---------- ---------- -NEALAYACM SAFIPKY--L YNFFLKDNSH VIQEYLTVF- ---------- ---------- -SQMIAFHDP ELSNHLNEIG ----------
TBC1D22A      VVFICEYIEA EEVDTVDVSG VPAEVLC--- NIEADTYWCM SKLLDG---- IQDNYTFAQP GIQMKVKML- ---------- ---------- -EELVSRIDE QVHRHLDQH- ----------
TBC1D22B      VVFLSEYVE- EDVENFDVTN LSQDMLR--- SIEADSFWCM SKLLDG---- IQDNYTFAQP GIQKKVKAL- ---------- ---------- -EELVSRIDE QVHNHFRRY- ----------
TBC1D13       YTFATDPNS- ---------- ------EWKE HAEADTFFCF TNLMAEIRDN FIKSLDDSQC GITYKMEKV- ---------- ---------- -YSTLKDKDV ELYLKLQEQ- ----------
```

```
              ....|....| ....|....| ....|....| ....|....| ....|....| ....|....| ....|....| ....|....| ....|....| ....|....| ....|....| ....|....|
                   365        375        385        395        405        415        425        435        445        455        465        475
TBC1D15       --DSGYLYFC FRWLLIRFKR EFS------- FLDILRLWEV MWTE--LPCT NFHLLLCCAI LESEKQQI-M EKHYGFNEIL KHINELS--M KIDVEDILCK AEAISLQMVK CKELPQAVCE
TBC1D17       --DSGSLCFC FRWLLIWFKR EFP------- FPDVLRLWEV LWTG--LPGP NLHLLVACAI LDMERDTL-M LSGFGSNEIL KHINELT--M KLSVEDVLTR AEALHRQLTA CPELPHNVQE
TBC1D16       -EDGLQMLFC HRWLLLCFKR EFP------- EAEALRIWEA CWAHY--QTD YFHLFICVAI VAIYGDDV-I EQQLATDQML LHFGNLA--M HMNGELVLRK ARSLLYQFRL LPRIPCSLHD
RUTBC1        --DYTHFYFC YRWFLLDFKR ELL------- YEDVFAVWEV IWAARHISSE HFVLFIALAL VEAYREII-R DNNMDFTDII KFFNERA--E HHDAQEILRI ARDLVHKVQM LIENK-----
RUTBC2        --DYTHFYFC YRWFLLDFKR ELV------- YDDVFLVWET IWAAKHVSSA HYVLFIALAL VEVYRDII-L ENNMDFTDII KFFNEMA--E RHNTKQVLKL ARDLVYKVQT LIENK-----
TBC1D5        --EIAPQIYG LRWVRLLFGR EFP------- LQDLLVVWDA LFADG--LSL GLVDYIFVAM LLYIRDAL-I SSNYQTCLGL LMHYPFIG-D VHSLILKALF LRDPKRNPRP VTYQFHPNLD
TBC1D10A      --KIDPLLYM TEWFMCAFSR TLP------- WSSVLRVWDM FFCEG----V KIIFRVGLVL LKHALGSP-E KVKACQGQYE TIERLRS--L SPKIM-QEAF LVQEVVELPV TERQIEREHL
TBC1D10B      --RIDPVLYM TEWFMCIFAR TLP------- WASVLRVWDM FFCEG----V KIIFRVALVL LRHTLGSV-E KLRSCQGMYE TMEQLRN--L PQQCM-QEDF LVHEVTNLPV TEALIERENA
TBC1D10C      --GVGPLLYL PEWFLCLFAR SLP------- FPTVLRVWDA FLSEG----A RVLFRVGLTL VRLALGTA-E QRGACPGLLE TLGALRA--I PPAQL-QEEA FMSQVHSVVL SERDLQREIK
TBC1D3C       --DLCGQCSP LGCLIRILID GIS------- LGLTLRLWDV YLVEG----E QALMPITRIA FKVQQKRL-T KTSRCG--PW ARFCNRF--V DTWAR-DEDT VLKHLR---A SMKKLTRKKG
TBC1D3B       --DLCGQCSP LGCLIRILID GIS------- LGLTLRLWDV YLVEG----E QALMPITRIA FKVQQKRL-T KTSRCG--PW ARFCNRF--V DTWAR-DEDT VLKHLR---A SMKKLTRKQG
USP6          --GLCGQCAS LGCLLRNLID GIS------- LGLTLRLWDV YLVEG----E QVLMPITSIA LKVQQKRL-M KTSRCG--LW ARLRNQF--F DTWAM-NDDT VLKHLR---A STKKLTRKQG
RN-TRE        --EIYTSFYT MKWFFQCFLD RTP------- FTLNLRIWDI YIFEG----E RVLTAMSYTI LKLHKKHL-M KLSMEE--LV EFFQETL--A KDFFF-EDDF VIEQLQ---I SMTELKRAKL
TBC1D1        --EIGPSLYA APWFLTMFAS QFP------- LGFVARVFDM IFLQG----T EVIFKVALSL LGSHKPLI-L QHENLETIVD FIKSTLP--N LGLV--QMEK TINQVFEMDI A-KQLQAYEV
TBC1D4        --EISPSLYA APWFLTLFAS QFS------- LGFVARVFDI IFLQG----T EVIFKVALSL LSSQETLI-M ECESFENIVE FLKNTLP--D MNTS--EMEK IITQVFEMDI S-KQLHAYEV
RabGAP-1      --SLEAHMYA SQWFLTLFTA KFP------- LYMVFHIIDL LLCEG----I SVIFNVALGL LKTSKDDL-L LTD-FEGALK FFRVQLP--K RYRSEENAKK LMELACNMKI SQKKLKKYEK
TBC1D18       --NLEAHMYA SQWFLTLFTA KFP------- LCMVFHIIDL LLCEG----L NIIFHVALAL LKTSKEDL-L QAD-FEGALK FFRVQLP--K RYRAEENARR LMEQACNIKV PTKKLKKYEK
EVI5          --SFHTSMYA SSWFLTIFLT TFP------- LPIATRIFDI FMSEG----L EIVFRVGLAL LQMNQAEL-M QLD-MEGMLQ HFQKVIP--H QFDG--VPDK LIQAAYQVKY NSKKMKKLEK
EVI5L         --SFHTSMYA SSWFLTLFLT TFP------- LPVATRVFDI FMYEG----L EIVFRVGLAL LQVNQAEL-M QLD-MEGMSQ YFQRVIP--H QFDS--CPDK LVLKAYQVKY NPKKMKRLEK
TBC1D14       --NLTPDIYL IDWIFTLYSK SLP------- LDLACRIWDV FCRDG----E EFLFRTALGI LKLFEDIL-T KMD------F IHMAQFL--T RLPEDLPAEE LFASIATIQM QSRNKKWAQV
TBC1D12       --SLTPDIYL IDWIFTLYSK SLP------- LDLACRVWDV FCRDG----E EFLFRTGLGI LRLYEDIL-L QMD------F IHIAQFL--T KLPEDITSEK LFSCIAAIQM QNSTKKWTQV
TBC1D2A       --HVDLSLVT FNWFLVVFAD SLI------- SNILLRVWDA FLYEG----T KVVFRYALAI FKYNEKEI-L RLQNGLEIYQ YLRFFTK--T ISNSRKLMNI AFNDMNPFRM KQLRQLRMVH
TBC1D2B       --KVDYTLIT FNWFLVVFVD SVV------- SDILFKIWDS FLYEG----P KVIFRFALAL FKYKEEEI-L KLQDSMSIFK YLRYFTR--T ILDARKLISI SFGDLNPFPL RQIRNRRAYH
RUTBC3        --DIELSLIT LHWFLTAFAS VVD------- IKLLLRIWDL FFYEG----S RVLFQLTLGM LHLKEEEL-I QSENSASIFN TLSDIPS--Q MEDAELLLGV AMRLAGSLTD VAVETQRRKH
TBC1D6        ---VLWTLLV SRWFICLFVD ILP------- VETVLRIWDC LFNEG----S KIIFRVALTL IKQHQELI-L EATSVPDICD KFKQITKGSF VMECHTFMQK IFSEPGSLSM ATVAKLRESC
TBC1D9A       --GV-ISTIS LSWFLTLFLS VMP------- FESAVVVVDC FFYEG----I KVIFQLALAV LDANVDKL-L NCKDDGEAMT VLGRYLD--S VTNKDSTLPP IPHLHSLLSD DVEPYPEVDI
TBC1D9B       --GV-ISSIS LSWFLTLFLS VMP------- FESAVVIVDC FFYEG----I KVILQVALAV LDANMEQL-L GCSDEGEAMT MLGRYLD--N VVNKQSVSPP IPHLRALLSS SDDPPAEVDI
TBC1D8B       --TF-FSSVS LSWFLTLFIS VLP------- IESAVNVVDC FFYDG----I KAILQLGLAI LDYNLDKL-L TCKDDAEAVT ALNRFFD--N VTNKDSPLPS NVQQGSNVSD EKTSHTRVDI
TBC1D8        --SA-LASVS LSWFLTLFLS IMP------- LESAVNVVDC FFYDG----I KAIFQLGLAV LEANAEDL-C SSKDDGQALM ILSRFLD--H IKNEDSPGPP VGSHHAFFSD DQEPYPVTDI
TBC1D30       YEPPLTNVFT MQWFLTLFAT CLP------- NQTVLKIWDS VFFEG----S EIILRVSLAI WAKLGEQI-E CCETADEFYS TMGRLTQEML ENDLLQSHEL MQTVYSMAPF PFPQLAELRE
TBC1D7        ---AAPKLPY DLWFKRCFAG CLP------- ESSLQRVWDK VVSGS----C KILVFVAVEI LLTFKIKV-M ALNSAEKITK FLENIPQ--- -----DSSDA IVSKAIDLWH KHCGTPVHSS
TBC1D20       VLDCDCDMAS VHHLLSQIPQ DLPY------ ETLISRAGDL FVQFP----P SELAREAAAQ QQAERTAA-S TFKDFELASA QQRPDMVLRQ RFRGLLRPED RTKDVLTKPR TNRFVKLAVM
TBC1D24       -FALCQPLLL FSSLQHGYSL ARFY------ FQCEGHEPTL LLIKT---TQ KEVCGAYLST DWSERNKFGG KLGFFGTGEC FVFRLQPEVQ RYEWVVIKHP ELTKPPPLMA AEPTAPLSHS
TBC1D23       --HWIASTSG SRSSINSVDG ESPNGSSDRG MKSLVNKMTV ALKTK----S VNVREKVISF IENTSTPVDR HVSSSDRVGK PYRGVKPVFS IGDEEEYDTD EIDSSSMSDD DRKEVVNIQT
TBC1D19       LYQIFREMYV RFFFRLHSIS SHPSG----- IVSLCLLFET LLQTY---LP QLFYHLREIG AQPLRISFKW MVRAFSGYLA TDQLLLLWDR ILGYNSLEIL AVLAAAVFAF RAVNLMEVTS
TBC1D21       --GAGAVQSL FPWFCFCFQR AFKS------ FDDVWRLWEV LLTGKP--CR NFQVLVAYSM LQMVREQV-L QESMGGDDIL LACNNLI--- DLDADELISA ACVVYAELIQ KDVPQTLKDF
TBCK          ---FIPDLYA IPWFLTMFTH VFP------- LHKIFHLWDT LLLGN----S SFPFCIGVAI LQQLRDRL-L ANGFNECILL FSDLPEIDIE RCVRESINLF CWTPKSATYR QHAQPPKPSS
TBC1D22A      --EVRYLQFA FRWMNNLLMR EVP------- LRCTIRLWDT YQSEP-DGFS HFHLYVCAAF LVRWRKEI-L EE-KDFQELL LFLQNLP--T AHWDDEDISL LLAEAYRLKF AFADAPNHYK
TBC1D22B      --EVEYLQFA FRWMNNLLMR ELP------- LRCTIRLWDT YQSEP-EGFS HFHLYVCAAF LIKWRKEI-L DE-EDFQGLL MLLQNLP--T IHWGNEEIGL LLAEAYRLKY MFADAPNHYR
TBC1D13       --NIKPQFFA FRWLTLLLSQ EFL------- LPDVIRIWDS LFADD--NRF DFLLLVCCAM LMLIREQL-L EGDFTVNMRL LQDYPIT--- ------DVCQ ILQKAKELQD SK--------
```

```
               ....|....| ....|....| ....|....| ....|....| ....|....| ....|....| ....|....| ....|....| ....|....| ....|....| ....|....| ....|....|
                   485        495        505        515        525        535        545        555        565        575        585        595
TBC1D15        ILGLQGSEVT TPDSDVGEDE NV-------- ---------- ---------- ---------- ---------- ---------- ---------- ---------- ---------- ----------
TBC1D17        ILGLAPPAEP HSPSPTASPL PLSPTRA--- ---------- ---------- ---------- ---------- ---------- ---------- ---------- ---------- ----------
TBC1D16        LCKLCGSGMW DSGSMPAVEC TGHHPGSES- ---------- ---------- ---------- ---------- ---------- ---------- ---------- ---------- ----------
RUTBC1         ---------- ---------- ---------- ---------- ---------- ---------- ---------- ---------- ---------- ---------- ---------- ----------
RUTBC2         ---------- ---------- ---------- ---------- ---------- ---------- ---------- ---------- ---------- ---------- ---------- ----------
TBC1D5         YYKARGADLM NKSRTNAKGA PLNINKVSNS LINF------ ---------- ---------- ---------- ---------- ---------- ---------- ---------- ----------
TBC1D10A       IQLRRWQETR GELQCR---- SPPRLHGAKA I--------- ---------- ---------- ---------- ---------- ---------- ---------- ---------- ----------
TBC1D10B       AQLKKWRETR GELQYR---- PSRRLHGSRA I--------- ---------- ---------- ---------- ---------- ---------- ---------- ---------- ----------
TBC1D10C       AQLAQLPDSA PGPPPR---- PQVRLAGAQA I--------- ---------- ---------- ---------- ---------- ---------- ---------- ---------- ----------
TBC1D3C        DLPPPAKPEQ GSSASR---- PVPASRGGKT LCKGDRQA-- ---------- ---------- ---------- ---------- ---------- ---------- ---------- ----------
TBC1D3B        DLPPPAKPEQ GSSASR---- PVPASRGRK- ---------- ---------- ---------- ---------- ---------- ---------- ---------- ---------- ----------
USP6           DLPPPAKREQ GSLAPR---- PVPASRGGKT L--------- ---------- ---------- ---------- ---------- ---------- ---------- ---------- ----------
RN-TRE         DLPEPGKEDE YPKKPLGQLP PELQSWGVHH LSN------- ---------- ---------- ---------- ---------- ---------- ---------- ---------- ----------
TBC1D1         EYHVLQEELI DSS-PLSDNQ RMDKLEKTNS SLRKQNL--- ---------- ---------- ---------- ---------- ---------- ---------- ---------- ----------
TBC1D4         EYHVLQDELQ ESSYSCEDSE TLEKLERANS QLKRQNM--- ---------- ---------- ---------- ---------- ---------- ---------- ---------- ----------
RabGAP-1       EYHTMREQQ- -----AQQED PIERFERENR RL-------- ---------- ---------- ---------- ---------- ---------- ---------- ---------- ----------
TBC1D18        EYQTMRESQ- -----LQQED PMDRYKFVYL ---------- ---------- ---------- ---------- ---------- ---------- ---------- ---------- ----------
EVI5           EYTTIKTKE- -----MEEQV EIKRLRTENR LLKQRI---- ---------- ---------- ---------- ---------- ---------- ---------- ---------- ----------
EVI5L          EYAAMKSKE- -----MEEQI EIKRLRTENR LLKQRI---- ---------- ---------- ---------- ---------- ---------- ---------- ---------- ----------
TBC1D14        LTALQKDSRE MEK-GSPSLR H--------- ---------- ---------- ---------- ---------- ---------- ---------- ---------- ---------- ----------
TBC1D12        FASVMKDIKE GDKNSSPALK S--------- ---------- ---------- ---------- ---------- ---------- ---------- ---------- ---------- ----------
TBC1D2A        RERLEAELRE LEQLKAEYLE RRASRRRAVS EGCAS----- ---------- ---------- ---------- ---------- ---------- ---------- ---------- ----------
TBC1D2B        LEKVRLELTE LEAIREDFLR ERDTSP---D KGELV----- ---------- ---------- ---------- ---------- ---------- ---------- ---------- ----------
RUTBC3         LAYLIADQGQ LLGAGTLTNL SQVVRRRTQR RKST------ ---------- ---------- ---------- ---------- ---------- ---------- ---------- ----------
TBC1D6         RARLLAQG-- ---------- ---------- ---------- ---------- ---------- ---------- ---------- ---------- ---------- ---------- ----------
TBC1D9A        FRLIRTSYEK FGTIRADLIE QMRFKQRLKV IQTL------ ---------- ---------- ---------- ---------- ---------- ---------- ---------- ----------
TBC1D9B        FELLKVSYEK FSSLRAEDIE QMRFKQRLKV IQSL------ ---------- ---------- ---------- ---------- ---------- ---------- ---------- ----------
TBC1D8B        TDLIRESNEK YGNIRYEDIH SMRCRNRLYV IQTL------ ---------- ---------- ---------- ---------- ---------- ---------- ---------- ----------
TBC1D8         SDLIRDSYEK FGDQSVEQIE HLRYKHRIRV LQGH------ ---------- ---------- ---------- ---------- ---------- ---------- ---------- ----------
TBC1D30        KYTYNITPFP ATVKPTSVSG RHSKARDS-- ---------- ---------- ---------- ---------- ---------- ---------- ---------- ---------- ----------
TBC1D7         ---------- ---------- ---------- ---------- ---------- ---------- ---------- ---------- ---------- ---------- ---------- ----------
TBC1D20        GLTVALGAAA LAVVKSALEW APKFQLQLFP ---------- ---------- ---------- ---------- ---------- ---------- ---------- ---------- ----------
TBC1D24        ASSDPADRLS PFLAARHFNL PSKTESMFMA GGSDCLIV-- ---------- ---------- ---------- ---------- ---------- ---------- ---------- ----------
TBC1D23        WINKPDVKHH FPCKEVKESG HMFPSHLLVT ATHMYCLREI VS-------- ---------- ---------- ---------- ---------- ---------- ----------
TBC1D19        LAAAEAVLAD LSTLKVMPLL QIFLFATVT- ---------- ---------- ---------- ---------- ---------- ---------- ---------- ---------- ----------
TBC1D21        FL-------- ---------- ---------- ---------- ---------- ---------- ---------- ---------- ---------- ---------- ---------- ----------
TBCK           DSSGGRSSAP YFSAECPDPP KTDLS----- ---------- ---------- ---------- ---------- ---------- ---------- ---------- ---------- ----------
TBC1D22A       K--------- ---------- ---------- ---------- ---------- ---------- ---------- ---------- ---------- ---------- ---------- ----------
TBC1D22B       R--------- ---------- ---------- ---------- ---------- ---------- ---------- ---------- ---------- ---------- ---------- ----------
TBC1D13        ---------- ---------- ---------- ---------- ---------- ---------- ---------- ---------- ---------- ---------- ---------- ----------
```

Supplementary Figure 2: Alignment of TBC1D10 subfamily. Conserved amino acids are labelled in green. Amino acids that are identical in two members are labelled in yellow.

```
              ....|....| ....|....| ....|....| ....|....| ....|....| ....|....| ....|....| ....|....|
                   5         15         25         35         45         55         65         75
TBC1D10A      ---------- ---------- ---------- ---------- ---------- ---------- ---------- ----------
TBC1D10B      METGTAPLVA PPRRHGAPAA PSPPPRGSRA GPVVVVAPGP PVTTATSAPV TLVAPGEARP AWVPGSAETS APAPAPAPAP
TBC1D10C      ---------- ---------- ---------- ---------- ---------- ---------- ---------- ----------

              ....|....| ....|....| ....|....| ....|....| ....|....| ....|....| ....|....| ....|....|
                   85         95        105        115        125        135        145        155
TBC1D10A      ---------- ---------- ---------- ---------- ---------- ---------- ---------- ----------
TBC1D10B      APAVTGSTVV VLTLEASPEA PKPQLPSGPE SPEPAAVAGV ETSRALAAGA DSPKTEEARP SPAPGPGTPT GTPTRTPSRT
TBC1D10C      ---------- ---------- ---------- ---------- ---------- ---------- ---------- ----------

              ....|....| ....|....| ....|....| ....|....| ....|....| ....|....| ....|....| ....|....|
                  165        175        185        195        205        215        225        235
TBC1D10A      ---------- ---------- ---------- ---------- -MAKSNGEN- -GPRAPAAGE S--------- ----------
TBC1D10B      APGALTAKPP LAPKPGTTVA SGVTARSASG QVTGGHGAAA ATSASAGQAP EDPSGPGTGP SGTCEAPVAV VTVTPAPEPA
TBC1D10C      ---------- ---------- ---------- ---------- -MAQALGED- ---------- ---------- ----------

              ....|....| ....|....| ....|....| ....|....| ....|....| ....|....| ....|....| ....|....|
                  245        255        265        275        285        295        305        315
TBC1D10A      ---------- -----LSGTR ---------- --ESLAQGPD AATTDELSSL GSDSEANG-F AERRIDKFGF IVGSQGAEGA
TBC1D10B      ENSQDLGSTS SLGPGISGPR GQAPDTLSYL DSVSLMSGTL ESLADDVSSM GSDSEING-L ALRKTDKYGF LGGSQYSGSL
TBC1D10C      ---------- ---------- ---------- ----LVQPP- -ELQDDSSSL GSDSELSGPG PYRQADRYGF IGGSS-AEPG

              ....|....| ....|....| ....|....| ....|....| ....|....| ....|....| ....|....| ....|....|
                  325        335        345        355        365        375        385        395
TBC1D10A      LEEVPLEVLR QRESKWLDML NNWDKWMAKK HKKIRLRCQK GIPPSLRGRA WQYLSGGKVK LQQNPGKFDE LDMSPGDPKW
TBC1D10B      ESSIPVDVAR QRELKWLDMF SNWDKWLSRR FQKVKLRCRK GIPSSLRAKA WQYLSNSKEL LEQNPGKFEE LERAPGDPKW
TBC1D10C      PGHPPADLIR QREMKWVEMT SHWEKTMSRR YKKVKMQCRK GIPSALRARC WPLLCGAHVC QKNSPGTYQE LAEAPGDPQW

              ....|....| ....|....| ....|....| ....|....| ....|....| ....|....| ....|....| ....|....|
                  405        415        425        435        445        455        465        475
TBC1D10A      LDVIERDLHR QFPFHEMFVS RGGHGQQDLF RVLKAYTLYR PEEGYCQAQA PIAAVLLMHM PAEQAFWCLV QICEKYLPGY
TBC1D10B      LDVIEKDLHR QFPFHEMFAA RGGHGQQDLY RILKAYTIYR PDEGYCQAQA PVAAVLLMHM PAEQAFWCLV QICDKYLPGY
TBC1D10C      METIGRDLHR QFPLHEMFVS PQGHGQQGLL QVLKAYTLYR PEQGYCQAQG PVAAVLLMHL PPEEAFWCLV QICEVYLPGY

              ....|....| ....|....| ....|....| ....|....| ....|....| ....|....| ....|....| ....|....|
                  485        495        505        515        525        535        545        555
TBC1D10A      YSEKLEAIQL DGEILFSLLQ KVSPVAHKHL SRQKIDPLLY MTEWFMCAFS RTLPWSSVLR VWDMFFCEGV KIIFRVGLVL
TBC1D10B      YSAGLEAIQL DGEIFFALLR RASPLAHRHL RRQRIDPVLY MTEWFMCIFA RTLPWASVLR VWDMFFCEGV KIIFRVALVL
TBC1D10C      YGPHMEAVRL DAEVFMALLR RLLPHVHKHL QQVGVGPLLY LPEWFLCLFA RSLPFPTVLR VWDAFLSEGA RVLFRVGLTL

              ....|....| ....|....| ....|....| ....|....| ....|....| ....|....| ....|....| ....|....|
                  565        575        585        595        605        615        625        635
TBC1D10A      LKHALGSPEK VKACQGQYET IERLRSLSPK IMQEAFLVQE VVELPVTERQ IEREHLIQLR RWQETRGELQ CRSPPRLHGA
TBC1D10B      LRHTLGSVEK LRSCQGMYET MEQLRNLPQQ CMQEDFLVHE VTNLPVTEAL IERENAAQLK KWRETRGELQ YRPSRRLHGS
TBC1D10C      VRLALGTAEQ RGACPGLLET LGALRAIPPA QLQEEAFMSQ VHSVVLSERD LQREIKAQLA QLPDSAPGPP PRPQVRLAGA

              ....|....| ....|....| ....|....| ....|....| ....|....| ....|....| ....|....| ....|....|
                  645        655        665        675        685        695        705        715
TBC1D10A      KAILDAEPGP RPALQPS--- ---------- ---------- -PSIRLPLDA PLPGSKAKPK ---------- ---PPKQAQK
TBC1D10B      RAIHEERRRQ QPPLGPSSSL LSLPGLKSRG SRAAGGAPSP PPPVRRASAG PAPGPVVTAE GLHPSLPSPT GNSTPLGSSK
TBC1D10C      QAIFEAQQLA G--------- ---------- ---------- ---VRRGAKP EVPRIVVQPP ---------- ------EEPR

              ....|....| ....|....| ....|....| ....|....| ....|....| ....|....| ....|....| ....|....|
                  725        735        745        755        765        775        785        795
TBC1D10A      EQRKQMKGRG QLEKPPAPNQ AMVVAAAGDA CPPQHVPPKD SAPKDSAPQD LAPQVS---- ---------- AHHRSQESLT
TBC1D10B      ETRKQEKERQ KQEKERQKQE KEREKERQKQ EKEREKQEKE REKQEKERQK QEKKAQGRKL SLRRKADGPP GPHDGGDRPS
TBC1D10C      PPRRKPQTRG ------KTFH GLLTRARG-- -PPIEGPPR- ---------- ---------- ---------- -PQRG-----

              ....|....
                  805
TBC1D10A      SQESEDTYL
TBC1D10B      AEARQDAYF
TBC1D10C      STSFLDTRF
```

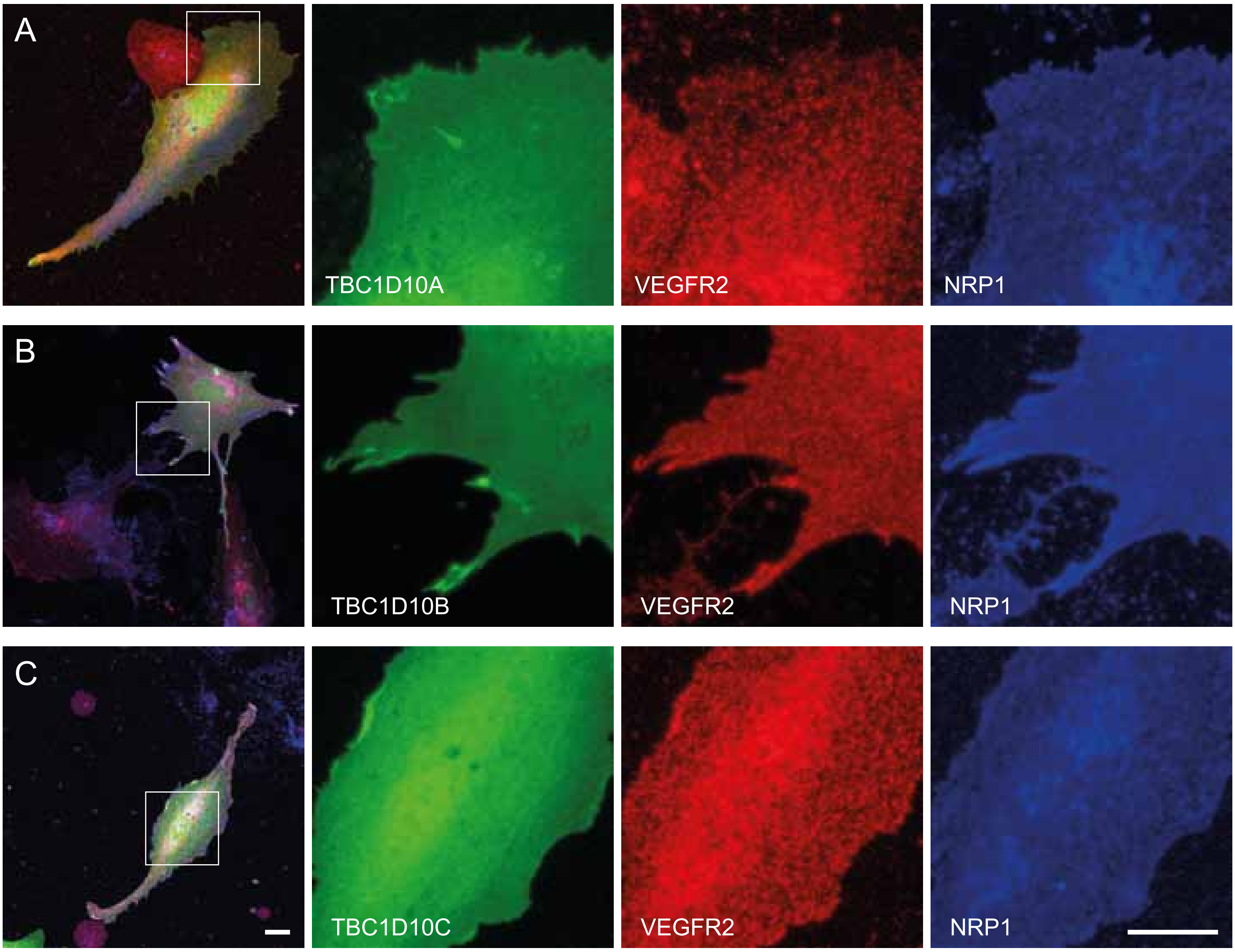

Supplementary Figure 3: VEGFR2, NRP1, and TBC1D10A-C localize to the plasma membrane in unstimulated PAE cells. PAE cells stably expressing VEGFR2 and NRP1 were transiently transfected with TBC1D10A (A), TBC1D10B (B), and TBC1D10C (C). Cells were fixed without stimulation with VEGF-A165a and immunostained for VEGFR2 and NRP1. Scale bar: 10 µm.

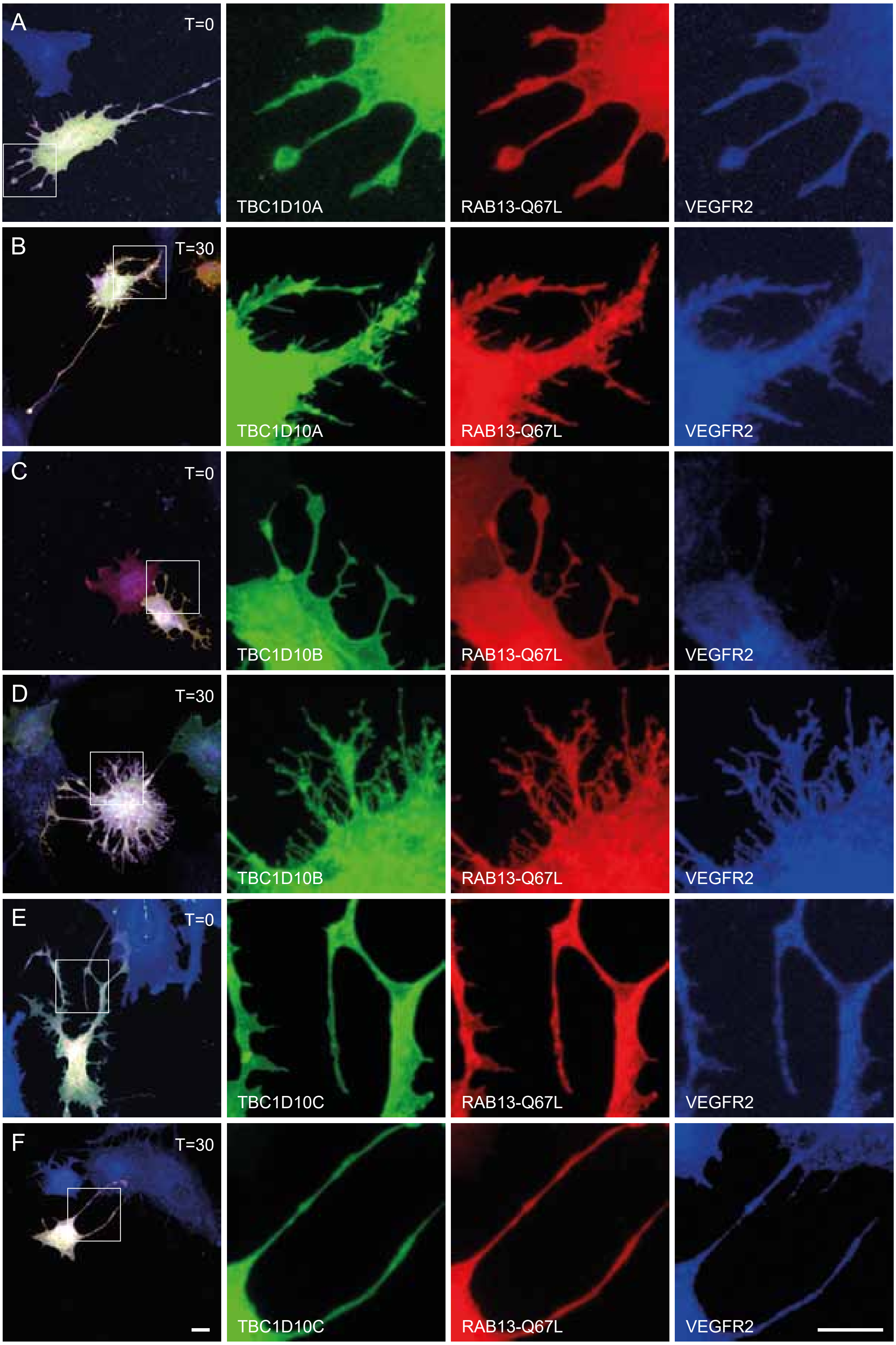

Coexpression of TBC1D10A/ B/ C with a constitutive active form of RAB13. (A) Coexpression of RAB13-Q67L with TBC1D10A induces filopodia in unstimulated cells that are positive for VEGFR2. (B) Thirty minutes after stimulation with VEGF A165a. (C) Coexpression of TBC1D10B with RAB13-Q67L. Filopodia in unstimulated cells are negative for VEGFR2. (D) Thirty minutes after stimulation with VEGF A165a, filopodia contain VEGFR2. (E) Coexpression of TBC1D10C with RAB13-Q67L. Filopodia in unstimulated cells are positive for VEGFR2. (F) Thirty minutes after stimulation with VEGF A165a, filopodia contain VEGFR2. Scale bar: 10 µm.
